# Control of RUNX-induced repression of Notch signaling by MLF and its partner DnaJ-1 during *Drosophila* hematopoiesis

**DOI:** 10.1371/journal.pgen.1006932

**Published:** 2017-07-25

**Authors:** Marion Miller, Aichun Chen, Vanessa Gobert, Benoit Augé, Mathilde Beau, Odile Burlet-Schiltz, Marc Haenlin, Lucas Waltzer

**Affiliations:** 1 Centre de Biologie du Développement (CBD), Centre de Biologie Intégrative (CBI), Université de Toulouse, CNRS, UPS, Toulouse, France; 2 Institut de Pharmacologie et de Biologie Structurale, Université de Toulouse, CNRS, UPS, Toulouse, France; New York University, UNITED STATES

## Abstract

A tight regulation of transcription factor activity is critical for proper development. For instance, modifications of RUNX transcription factors dosage are associated with several diseases, including hematopoietic malignancies. In *Drosophila*, Myeloid Leukemia Factor (MLF) has been shown to control blood cell development by stabilizing the RUNX transcription factor Lozenge (Lz). However, the mechanism of action of this conserved family of proteins involved in leukemia remains largely unknown. Here we further characterized MLF’s mode of action in *Drosophila* blood cells using proteomic, transcriptomic and genetic approaches. Our results show that MLF and the Hsp40 co-chaperone family member DnaJ-1 interact through conserved domains and we demonstrate that both proteins bind and stabilize Lz in cell culture, suggesting that MLF and DnaJ-1 form a chaperone complex that directly regulates Lz activity. Importantly, *dnaj-1* loss causes an increase in Lz^+^ blood cell number and size similarly as in *mlf* mutant larvae. Moreover we find that *dnaj-1* genetically interacts with *mlf* to control Lz level and Lz^+^ blood cell development *in vivo*. In addition, we show that *mlf* and *dnaj-1* loss alters Lz^+^ cell differentiation and that the increase in Lz^+^ blood cell number and size observed in these mutants is caused by an overactivation of the Notch signaling pathway. Finally, using different conditions to manipulate Lz activity, we show that high levels of Lz are required to repress *Notch* transcription and signaling. All together, our data indicate that the MLF/DnaJ-1-dependent increase in Lz level allows the repression of *Notch* expression and signaling to prevent aberrant blood cell development. Thus our findings establish a functional link between MLF and the co-chaperone DnaJ-1 to control RUNX transcription factor activity and Notch signaling during blood cell development *in vivo*.

## Author summary

Tight regulation of proteins level is required for proper development. Notably, the aberrant expression of key transcription factors or signaling pathway components controlling blood cell development contributes to hematological diseases such as leukemia. In this report, we use *Drosophila* as a model to study the function and mode of action of a family of conserved but poorly characterized proteins implicated in leukemia called Myeloid Leukemia Factors (MLF). By combining proteomic, transcriptomic and genetic approaches, we show that *Drosophila* MLF acts in concert with an Hsp40 co-chaperone to control the level and activity of a RUNX transcription factor and therefore RUNX^+^ blood cell number and differentiation. Furthermore, we show that RUNX dosage directly impinges on the activity of the Notch signaling pathway, which is critical for RUNX^+^ cell survival and differentiation, by regulating the transcription of the Notch receptor. These findings shed light on a new mode of regulation of RUNX level and Notch activity to prevent abnormal blood cell accumulation, which could be involved in leukemogenesis.

## Introduction

Proper blood cell development requires the finely tuned regulation of transcription factors and signaling pathways activity. Consequently mutations affecting key regulators of hematopoiesis such as members of the RUNX transcription factor family or components of the Notch signaling pathway are associated with several blood cell disorders including leukemia [1, 2]. Also, leukemic cells often present recurrent chromosomal rearrangements that participate in malignant transformation by altering the function of these factors [3]. The functional characterization of these genes is thus of importance not only to uncover the molecular basis of leukemogenesis but also to decipher the regulatory mechanisms controlling normal blood cell development. *Myeloid Leukemia Factor 1* (*MLF1*) was identified as a target of the t(3;5)(q25.1;q34) translocation associated with acute myeloid leukemia (AML) and myelodysplastic syndrome (MDS) more than 20 years ago [4]. Further findings suggested that *MLF1* could act as an oncogene [5–8] or a tumor suppressor [9] depending on the cell context and it was shown that MLF1 overexpression either impairs cell cycle exit and differentiation [10], promotes apoptosis [11, 12], or inhibits proliferation [13, 14] in different cultured cell lines. Yet, its function and mechanism of action remain largely unknown.

MLF1 is the founding member of a small evolutionarily conserved family of nucleo-cytoplasmic proteins present in all metazoans but lacking recognizable domains that could help define their biochemical activity [15]. Whereas vertebrates have two closely related MLF paralogs, *Drosophila* has a single *mlf* gene encoding a protein that displays around 50% identity with human MLF in the central conserved domain [16, 17]. In the fly, MLF was identified as a partner of the transcription factor DREF (DNA replication-related element-binding factor) [16], for which it acts a co-activator to stimulate the JNK pathway and cell death in the wing disc [18]. MLF has been shown to bind chromatin [18–20], as does its mouse homolog [21], and it can either activate or repress gene expression by a still unknown mechanism [18, 20]. MLF also interacts with Suppressor of Fused, a negative regulator of the Hedgehog signaling pathway [19], and, like its mammalian counterpart [13], with Csn3, a component of the COP9 signalosome [22], but the functional consequences of these interactions remain elusive. Interestingly the overexpression of *Drosophila* MLF or that of its mammalian counterparts can suppress polyglutamine-induced cytotoxicity in fly and in cellular models of neurodegenerative diseases [17, 23–25]. Moreover phenotypic defects associated with MLF loss in *Drosophila* can be rescued by human MLF1 [17, 26]. Thus MLF function seems conserved during evolution and *Drosophila* appears to be a genuine model organism to characterize MLF proteins [15].

Along this line, we recently analyzed the role of MLF during *Drosophila* hematopoiesis [26]. Indeed, a number of proteins regulating blood cell development in human, such as RUNX and Notch, also control *Drosophila* blood cell development [27]. In *Drosophila*, the RUNX factor Lozenge (Lz) is specifically expressed in crystal cells and it is absolutely required for the development of this blood cell lineage [28]. Crystal cells account for ±4% of the circulating larval blood cells; they are implicated in melanization, a defense response related to clotting, and they release their enzymatic content in the hemolymph by bursting [27]. The Notch pathway also controls the development of this lineage: it is required for the induction of Lz expression and it contributes to Lz^+^ cell differentiation as well as to their survival by preventing their rupture [28–31]. Interestingly, our previous analysis revealed a functional and conserved link between MLF and RUNX factors [26]. In particular, we showed that MLF controls Lz activity and prevents its degradation in cell culture and that the regulation of Lz level by MLF is critical to control crystal cell number *in vivo* [26]. Intriguingly, although Lz is required for crystal cell development, *mlf* mutation causes a decrease in Lz expression but an increase in crystal cell number. In human, the deregulation of RUNX protein level is associated with several pathologies. For instance haploinsufficient mutations in *RUNX1* are linked to MDS/AML in the case of somatic mutations, and to familial platelet disorders associated with myeloid malignancy for germline mutations [1]. In the opposite, RUNX1 overexpression can promote lymphoid leukemia [32, 33]. Understanding how the level of RUNX protein is regulated and how this affects specific developmental processes is thus of particular importance.

To better characterize the function and mode of action of MLF in *Drosophila* blood cells, we used proteomic, transcriptomic and genetic approaches. In line with recent findings [20], we found that MLF binds DnaJ-1, a HSP40 co-chaperone, as well as the HSP70 chaperone Hsc70-4, and that both of these proteins are required to stabilize Lz. We further show here that MLF and DnaJ-1 interact together but also with Lz *via* conserved domains and that they regulate Lz-induced transactivation in a Hsc70-dependent manner in cell culture. In addition, using a null allele of *dnaj-1*, we show that it controls Lz^+^ blood cell number and differentiation as well as Lz activity *in vivo* in conjunction with *mlf*. Notably, we found that *mlf* or *dnaj-1* loss leads to an increase in Lz^+^ cell number and size due to the over-activation of the Notch signaling pathway. Interestingly, our results indicate that high levels of Lz are required to repress Notch expression and signaling. We thus propose a model whereby MLF and DnaJ-1 control Lz^+^ blood cell growth and number by promoting Lz accumulation, which ultimately turndowns Notch signaling. These findings thus establish a functional link between the MLF/Dna-J1 chaperone complex and the regulation of a RUNX-Notch axis required for blood cell homeostasis *in vivo*.

## Results

### MLF interacts with DnaJ-1 via conserved domains

**Fig 1:**
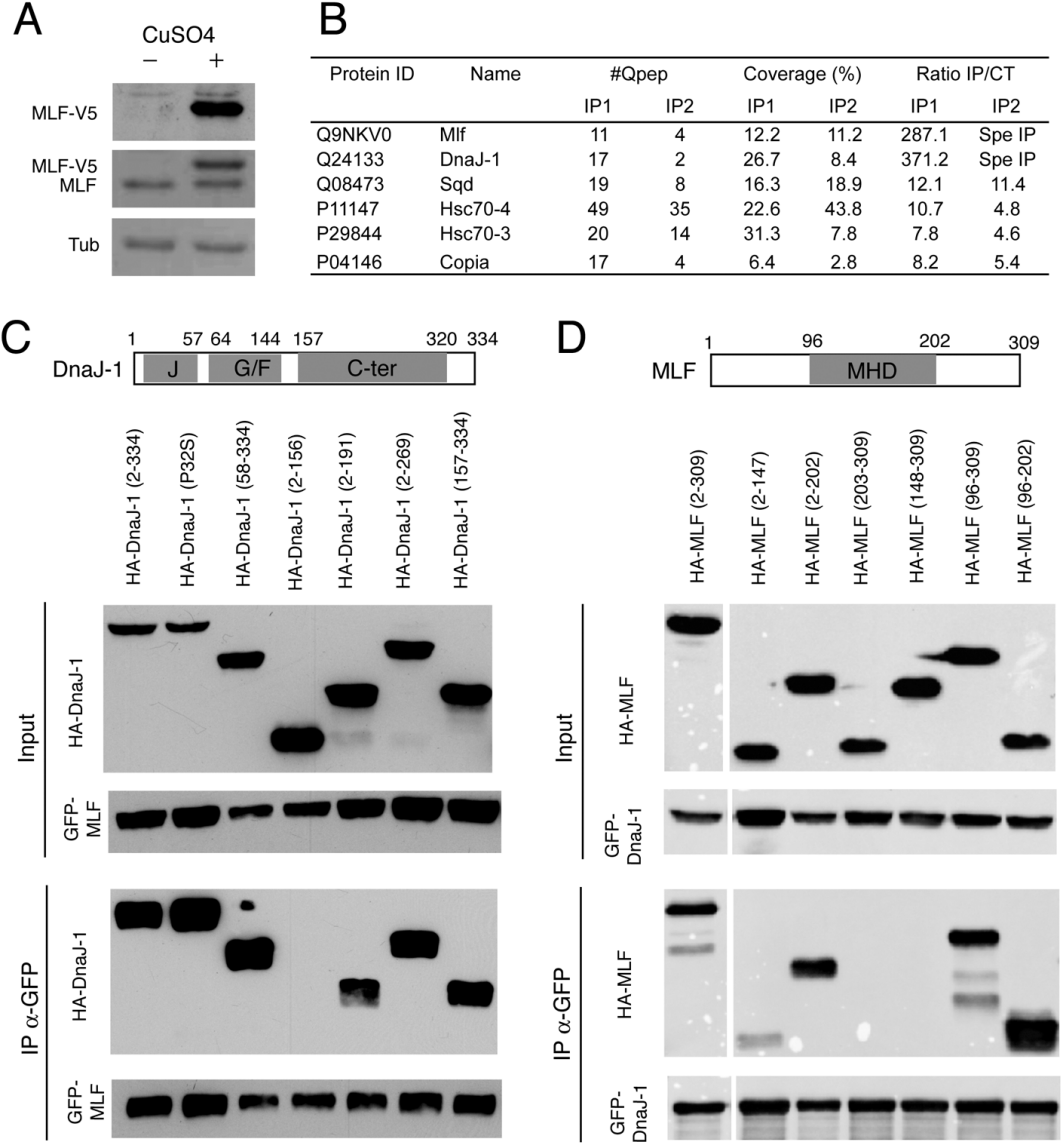
MLF and the co-chaperone DnaJ-1 interact *via* conserved domains. (A) Western blots showing MLF and MLF-V5 expression in Kc167 cells stably transfected with the copper-inducible pMT-MLF-V5 expression vector and treated or not with 50 μm CuSO_4_ for 24h. Tubulin (Tub) was used as an internal loading control. (B) Proteins identified by mass spectrometry from CuSO_4_-induced Kc167-pMT-MLF-V5 cells using anti-V5 antibody coupled to sepharose (IP1) or magnetic (IP2) beads for purification. The number of quantified peptides (#Qpep), sequence coverage and fold enrichment in comparison to control (parental Kc167 cells) are indicated for each experiment. Spe IP: not detected in control condition. (C, D) Schematic representation of DnaJ-1 (C) and MLF protein domains (D) and Western blots showing the results of immunoprecipitation experiments against GFP performed in Kc167 cells transfected with expression vectors for GFP-MLF and various HA-DnaJ-1 mutants (C) or GFP-DnaJ-1 and different HA-MLF mutants (D). Conserved domains are highlighted in grey. J: J-domain. G/F: glycine/phenylalanine-rich region. C-ter: C-terminal domain. MHD: MLF homology domain.

To better characterize the molecular mode of action of MLF, we sought to identify its partners. Accordingly, we established a *Drosophila* Kc167 cell line expressing a V5-tagged version of MLF close to endogenous levels in a copper-inducible manner (Fig 1A). After anti-V5 affinity purification from whole cell extracts of control or MLF-V5-expressing cells, isolated proteins were identified by mass spectrometry. Five proteins reproducibly co-purified with MLF and were either absent or at more than 4 fold lower levels in each control purification (Fig 1B): the Hsp40 co-chaperone DnaJ-1 (also known as DROJ1; [34]), the constitutively expressed Hsp70 chaperones Hsc70-4 and Hsc70-3, the RNA binding protein Squid (Sqd), and the retrotransposon-encoded protein Copia. Of note, as this manuscript was in preparation, Dyer *et al*. also identified DnaJ-1 and Hsc70-4 as partners of MLF using a similar proteomic approach in the *Drosophila* S2 cell line [20]. Since DnaJ-1 was the strongest hit in our analysis, we focused on this candidate and we further characterized its interaction with MLF as well as its function both in cell culture and *in vivo*.

First, we confirmed the interaction between MLF and DnaJ-1 by co-immunoprecipitation assays in Kc167 cells transfected with expression plasmids for tagged versions of these proteins using anti-tag antibodies (Fig 1C and 1D, and S1 Fig) or an anti-MLF antibody (S1C Fig). In addition, consistent with the hypothesis that these proteins interact in the cell, immunostainings showed that DnaJ-1 and MLF co-localize in the nuclei of Kc167 transfected cells (S1D Fig). Finally, we also observed a specific interaction between MLF and DnaJ-1 by *in vitro* GST pull down assays (S1E Fig).

We then mapped the domains required for the interaction between DnaJ-1 and MLF. Hsp40/DnaJ co-chaperones play a crucial role in the regulation of protein folding and degradation; they chiefly act by delivering substrates to Hsp70/DnaK chaperones and stimulating their ATPase activity [35, 36]. DnaJ-1 belongs to the DnaJB/class II subfamily of Hsp40/DnaJ proteins, which are characterized by an N-terminal J-domain required to stimulate Hsp70 ATPase activity (amino acids 4 to 57 in DnaJ-1), a central glycine/phenylalanine (G/F)-rich region (amino acids 64 to 144), and a conserved C-terminal region (amino acids 157 to 320) that contains the client proteins binding domain followed by a dimerization interface [36]. Immunoprecipitations of GFP-MLF expressed with different HA-tagged DnaJ-1 variants indicated that the DnaJ-1 C-terminal region mediates MLF binding (Fig 1C). In contrast, a point mutation (P32S) in the highly conserved HPD loop crucial for Hsc70 activation [36], deletion of the J-domain or deletion of the J and G/F domains did not affect the interaction between DnaJ-1 and GFP-MLF. MLF does not harbor characteristic domains apart from a central “MLF homology domain” (MHD, amino acids 96 to 202) conserved between MLF family members [15]. Using GFP-DnaJ-1 as bait and MLF deletion mutants as preys, we found that the MHD was sufficient for binding DnaJ-1, while MLF N- and C-terminal regions were dispensable (Fig 1D). Finally, consistent with the above results, the C-terminal region (amino acids 157 to 334) of DnaJ-1 bound to the MHD of MLF (S1F Fig). In sum, these data indicate that MLF and DnaJ-1 specifically bind each other through their conserved central and C-terminal region, respectively.

### MLF and DnaJ-1 interact with Lz and control its activity

**Fig 2:**
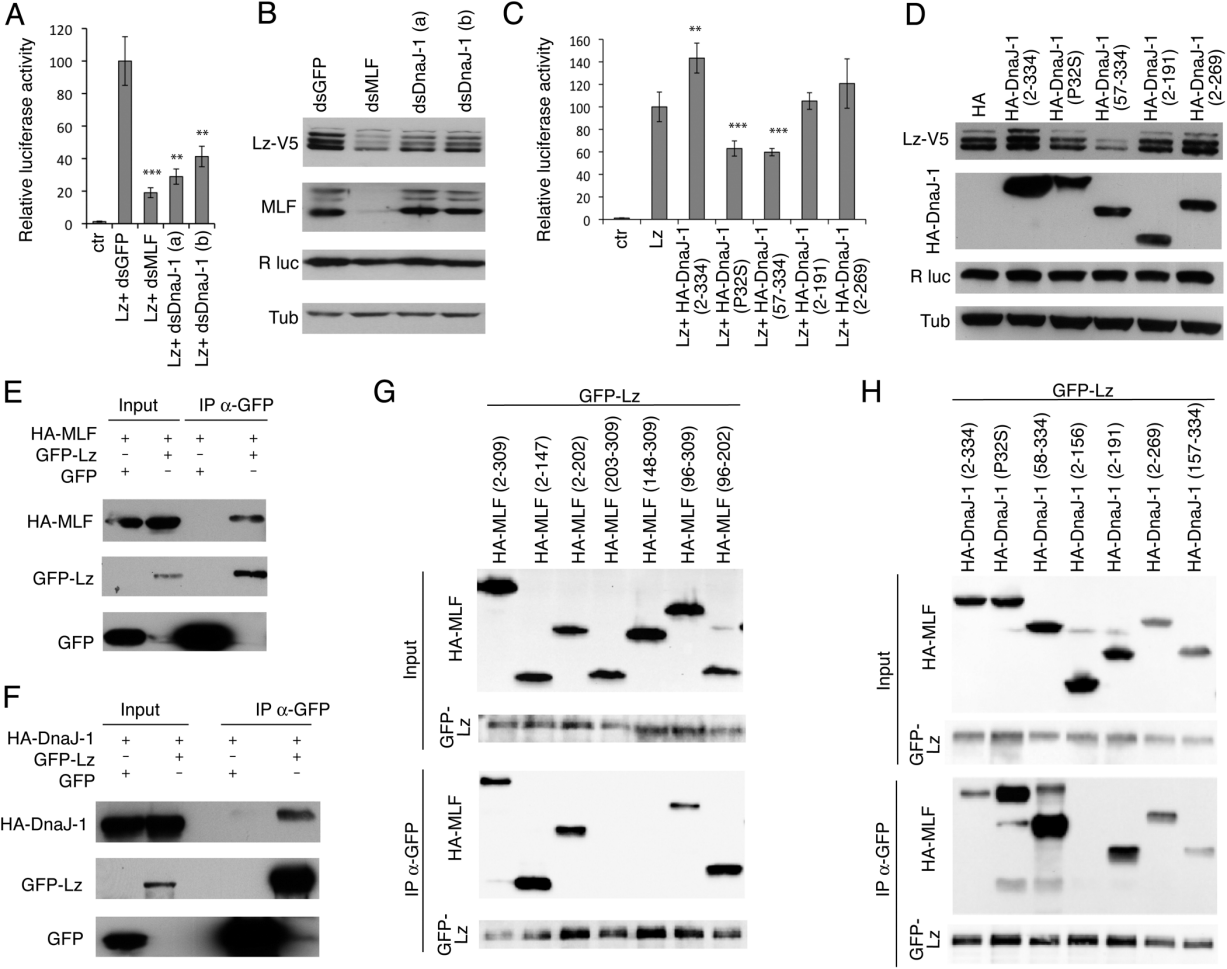
MLF and DnaJ-1 bind Lz and control its stability and activity. (A) Luciferase assays in Kc167 cells treated with the indicated dsRNA and transfected with 4xPPO2-Fluc reporter plasmid in the presence or not (ctr) of the pAc-Lz-V5 expression plasmid. pAc-Rluc was used as an internal normalization control. (B) Western blots showing Lz-V5, MLF, *Renilla* luciferase (R luc) and Tubulin (Tub) expression in Kc167 cells treated with the indicated dsRNA and cotransfected with pAc-Lz-V5 and pAc-Rluc expression vectors. (A, B) dsDnaJ-1 (a) and (b) correspond to two distinct dsRNAs targeting *dnaj-1*. Of note, the multiple bands for Lz are only observed using C terminally (V5) tagged versions of Lz and not with N terminally (GFP) tagged Lz; they likely represent internal translation initiation events. The multiple bands observed using a MLF antibody could represent different MLF protein isoforms as described in [17]. (C, D) Luciferase assays (C) and Western blots (D) performed on Kc167 cells transfected with the 4xPPO2-Fluc reporter plasmid and pAc-based expression plasmids for Lz and for different DnaJ-1 variants as indicated. Rluc and Tubulin were used as internal controls. (E, F) Western blots showing the results of immunoprecipitation experiments against GFP performed in Kc167 cells transfected with expression vectors for HA-MLF (E) or HA-DnaJ-1 (F) and GFP or GFP-Lz as indicated in the upper part of the panels. (G, H) Western blots showing the results of immunoprecipitation experiments against GFP performed in Kc167 cells transfected with expression vectors for GFP-Lz and various HA-MLF (G) or HA-DnaJ-1 (H) mutants. (A, C) For luciferase assays means and standard deviations of results from biological triplicates are shown. ***: p-value<0.001, **: p-value<0.01 (Students t-tests) as compared to Lz with dsGFP condition.

We have previously shown that MLF is required for Lz stability and transcriptional activity [26]. Interestingly, Dyer *et al*. reported that the knockdown of DnaJ-1 or of its chaperone partner Hsc70-4 leads to a destabilization of exogenously expressed Lz in S2 cells [20]. However, the relationships between DnaJ-1, MLF and Lz were not further explored. We thus asked whether DnaJ-1 also controls Lz activity. As shown in Fig 2A, transfection of a Lz expression plasmid in Kc167 cells induced a robust activation of the *4xPPO2-Fluc* reporter gene [37], which was significantly decreased when either *mlf* or *dnaj-1* expression was knocked down by dsRNA treatment. Consistent with previous results [20, 26], Western blot analyses showed that *mlf* and *dnaj-1* knockdowns caused a drop in Lz protein level (Fig 2B). Moreover, RT-qPCR experiments showed that *mlf* and *dnaj-1* knockdowns did not affect the expression of each other or decrease *lz* transcript level, while they did cause a significant reduction in the expression of Lz target gene *ppo2* (S2A–S2D Fig). Hence, like MLF, DnaJ-1 controls Lz protein stability and activity in Kc167 cells.

Next, we tested the effect of DnaJ-1 overexpression on Lz’s activity and protein level. Reminiscent of MLF [26], we observed that DnaJ-1 over-expression was associated with an increase in Lz-induced transactivation and Lz level (Fig 2C and 2D). The overexpression of C-terminally-truncated DnaJ-1 proteins did not affect Lz-induced transcription or its expression. In contrast, the overexpression of DnaJ-1 carrying the P32S point mutation or a deletion of its J-domain caused a decrease in Lz-induced transcription and a drop in Lz level (Fig 2C and 2D), suggesting that the activation of Hsc70 by DnaJ-1 is required for Lz’s stable expression and activity. In line with this hypothesis, knocking down Hsc70-4, which interacts with DnaJ-1 and MLF [20], caused a strong decrease in Lz-induced transactivation and a concomitant reduction in Lz protein level (S2E and S2F Fig). In sum, our results support the idea that MLF acts with DnaJ-1 in a Hsc70 chaperone complex to promote Lz stability and activity.

Given the impact of MLF and DnaJ-1 on Lz activity, we then asked whether these two proteins bind this RUNX transcription factor. Upon transfection of the corresponding expression plasmids, both HA-DnaJ-1 and HA-MLF were co-immunoprecipitated by GFP-tagged Lz but not by GFP alone (Fig 2E and 2F). Furthermore, *in vitro* translated Lz bound to *E*. *coli*-purified GST-MLF and GST-DnaJ-1 but not to GST alone in pull down assays (S2G Fig). Using different MLF variants in co-immunoprecipitation assays, we found that the N-terminal part of the MLF homology domain (amino acids 96 to 147) was crucial for the interaction with Lz (Fig 2G). Similarly the C-terminal domain of DnaJ-1 was required for binding Lz, while its J domain was dispensable (Fig 2H). Therefore it appears that MLF and DnaJ-1 interact with Lz through conserved domains and our results suggest that the MLF/DnaJ-1 complex regulates Lz stability and activity in Kc167 cells by binding it.

### DnaJ-1 acts with MLF to control Lz^+^ blood cell number and size *in vivo*

**Fig 3:**
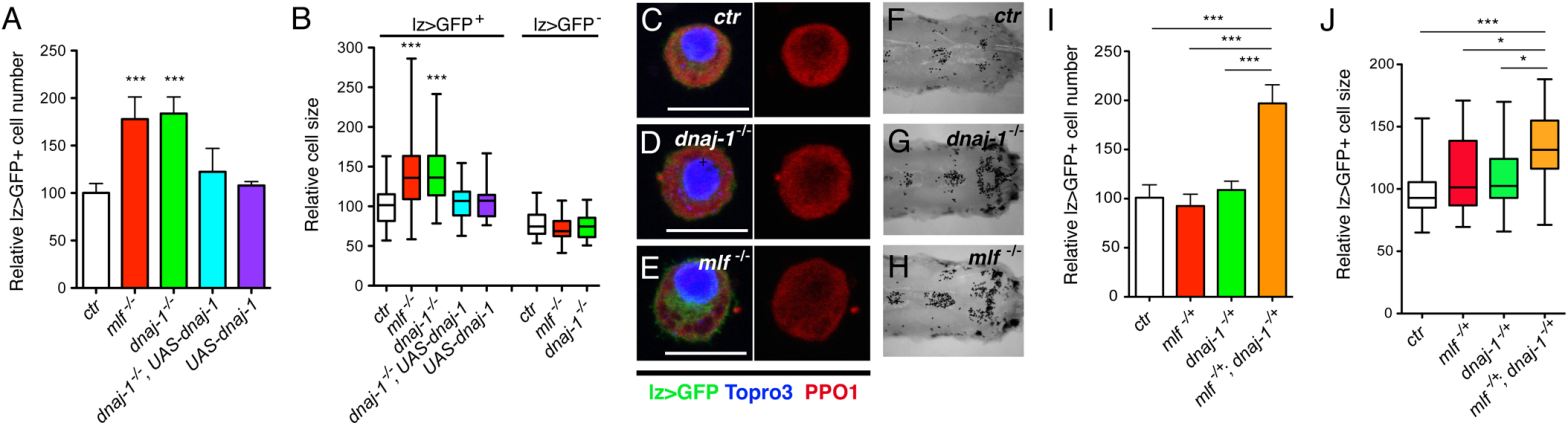
*dnaj-1* controls crystal cell development. (A, B) Quantification of circulating lz>GFP^+^ cell number (A) and lz>GFP^+^ or lz>GFP^-^ cell size (B) in *lz-GAL4*, *UAS-mCD8-GFP/+* third instar larvae of the indicated genotypes. (C-E) Fluorescent immunostainings of the crystal cell differentiation marker PPO1 in third instar lz>GFP^+^ hemocytes. The right panels show PPO1 immunostaining only. Nuclei were stained with Topro3. Scale bar: 10 μm. (F-H) Bright field images of the posterior segments of third instar larvae heat-treated at 65°C for 10 min to induce crystal cell melanization. (I, J) Relative lz>GFP^+^ blood cell number (I) and size (C) in *lz-GAL4*, *UAS-mCD8-GFP/+* third instar larvae of the indicated genotypes. (A, B, I, J) *:p-value<0.05, **: p-value<0.01 and ***: p-value<0.001 compared to control.

Since DnaJ-1 interacts with MLF and controls Lz level *ex vivo*, we then sought to analyze DnaJ-1 function in circulating larval crystal cells, whose proper development requires Lz stabilization by MLF [26]. Given that no mutant for *dnaj-1* was available, we used a CRISPR/Cas9 strategy to generate *dnaj-1* null alleles (S3 Fig) [38]. In the following experiments, we used an allelic combination between two mutant lines obtained from independent founder flies (*dnaj-1*^*A*^ and *dnaj-1*^*C*^), which harbor a complete deletion of the *dnaj-1* coding sequence (S3 Fig). Around 65% of the *dnaj-1*^A/C^ mutants reached the larval stage and 15% emerged as adult flies but they did not show obvious morphological defects. Reminiscent of *mlf* phenotypes [26], bleeding of third instar larvae revealed that *dnaj-1* mutants exhibited a ±1.8-fold increase in the number of circulating lz>GFP^+^ blood cells as compared to wild-type (Fig 3A). In addition, as in the *mlf* mutant, crystal cells from *dnaj-1* mutant larvae still expressed the differentiation marker PPO1 and were capable of melanization upon heat treatment (Fig 3C–3H). A closer examination also revealed the presence of unusually large lz>GFP^+^ cells in the *dnaj-1* mutant and quantitative analyses confirmed that *dnaj-1* loss caused a significant increase in lz>GFP^+^ cell size whereas lz>GFP^-^ cells were unaffected (Fig 3B). Interestingly, a similar phenotype is observed in *mlf* mutant larvae (Fig 3B), suggesting that both genes not only control crystal cell number but also their differentiation (see below). Importantly, lz>GFP^+^ cell number and size was restored to wild-type when DnaJ-1 was re-expressed in the crystal cell lineage of *dnaj-1*^*A/C*^ mutant larvae using the *lz-GAL4* driver (Fig 3A and 3B). This demonstrates not only that these phenotypes are specifically caused by the *dnaj-1* mutation, but also that DnaJ-1 acts cell autonomously and after the onset of *lz* expression in the crystal cell lineage. Of note, we did not observe a rescue when we expressed a DnaJ-1 protein lacking its J-domain, suggesting that the interaction with Hsp70 chaperones is critical for the function of DnaJ-1 in the crystal cell lineage (S3C and S3D Fig). Furthermore, the increase in crystal cell number and size was also observed when we monitored crystal cell presence by immunostaining against PPO1 in larvae carrying a *dnaj-1*^*A*^ or *dnaj-1*^*C*^ homozygous mutation or over a deficiency uncovering the *dnaj-1* locus (S3E and S3F Fig). Overall, these results demonstrate that, like *mlf*, *dnaj-1* controls circulating larval lz>GFP^+^ cell number and size.

Since MLF and DnaJ-1 bind to each other, we tested whether they genetically interacted to regulate crystal cell development. While heterozygous mutation in either *mlf* or *dnaj-1* did not significantly alter circulating lz>GFP^+^ cell number or size, *mlf*^*ΔC1*^*/+*,*dnaj-1*^*A*^*/+* transheterozygote larvae displayed a significant increase of both parameters (Fig 3I and 3J). We thus conclude that DnaJ-1 and MLF act together to control crystal cell development. In sum, these results reveal a functional interaction between MLF and DnaJ-1 *in vivo*.

### High levels of MLF prevent Lz degradation in the absence of DnaJ-1

**Fig 4:**
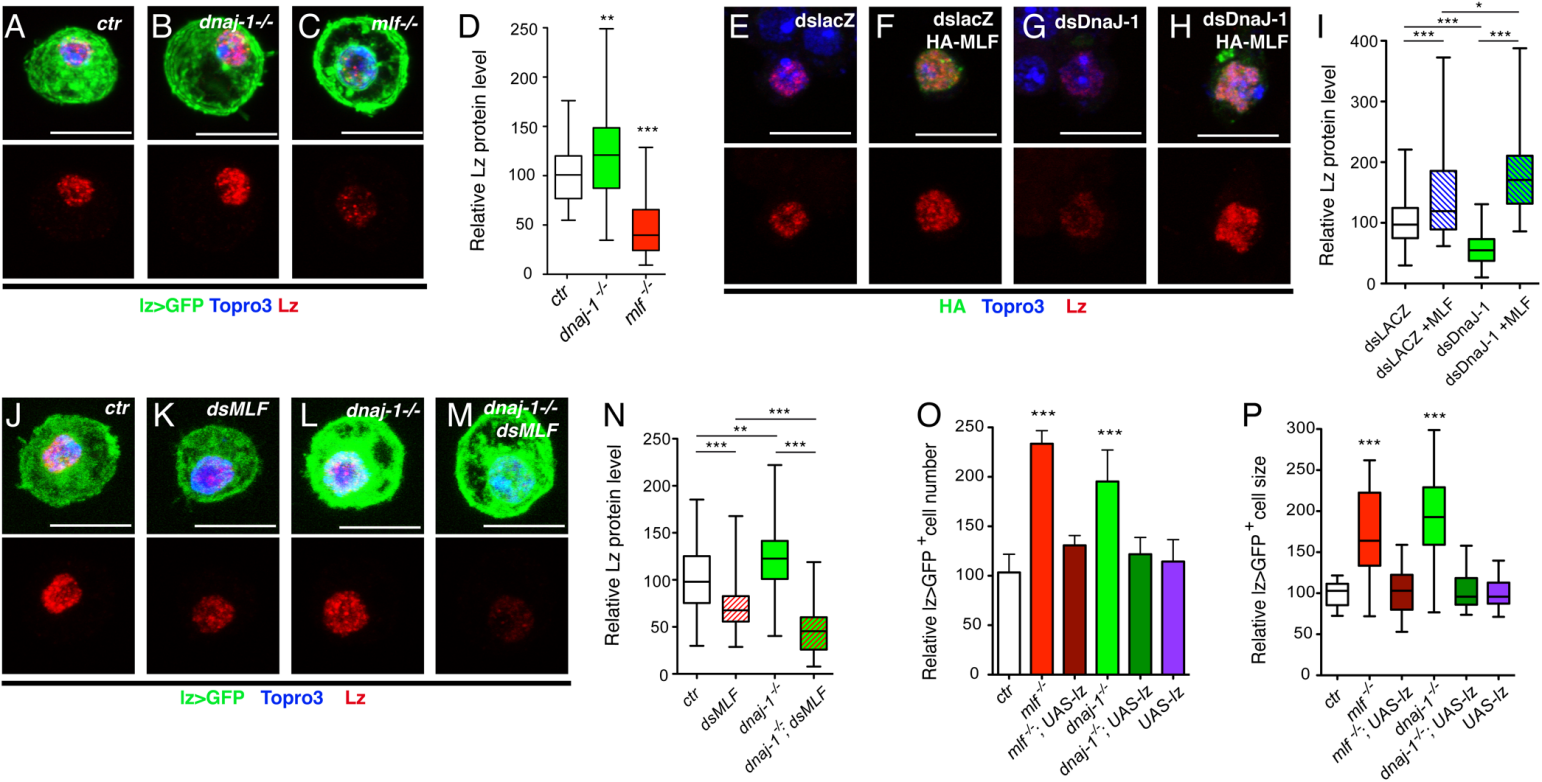
High levels of MLF prevent Lz degradation in the absence of DnaJ-1. (A-C) Fluorescent immunostainings of Lz in circulating blood cells from *lz-GAL4*, *UAS-mCD8-GFP/+* control (A), *dnaj1*^*-/-*^ (B) and *mlf*^*-/-*^ (C) third instar larvae. (D) Corresponding quantifications of Lz protein level. (E-H) Immunostainings against Lz (red) and HA-MLF (green) in Kc167 cells treated with the indicated dsRNA and transfected with pAc-Lz-V5 alone (E, G) or in combination with pAc-3HA-MLF (F, H). (I) Corresponding quantification of Lz levels in Kc167 cells. (J-M) Immunostainings against Lz in circulating blood cells from *lz-GAL4*, *UAS-mCD8-GFP/+* control (J), *UAS-dsMLF* (K), *dnaj1*^*-/-*^ (L) and *UAS-dsMLF*; *dnaj1*^*-/-*^ (M) third instar larvae. (N) Corresponding quantification of Lz protein levels in lz>GFP^+^ larval blood cells. (A-C, E-H, J-M) Nuclei were stained with Topro3. Lz staining only is shown in the lower panels. Scale bar: 10 μm. (O, P) Relative lz>GFP^+^ blood cell number (O) and size (P) in *lz-GAL4*, *UAS-mCD8-GFP/+* third instar larvae of the indicated genotypes. (D, I, N-P) *: p-value<0.05, **: p-value<0.01, ***: p-value<0.001.

Next we assessed whether DnaJ-1 affects Lz stability *in vivo* as it does in cell culture. Unexpectedly, immunostaining against Lz did not reveal a decrease in Lz expression in *dnaj-1* mutant crystal cells while the level of Lz was clearly lower in the *mlf* mutant (Fig 4A–4C). Actually quantitative analyses revealed a slight (30%) but significant (*p* = 0.006) increase in Lz level in *dnaj-1* mutant as compared to wild-type, whereas Lz level dropped by more than 2 folds in *mlf* mutant (Fig 4D). Thus, unlike *mlf*, *dnaj-1* loss is not sufficient to destabilize Lz *in vivo*. We then tried to understand the reason for this discrepancy. One potentially important difference between Kc167 cells, in which DnaJ-1 is required to stabilize Lz, and crystal cells, in which it is not, is MLF expression. Indeed, in Kc167 cells, MLF is mainly detected in the cytoplasm and is present at low levels in the nucleus (S4A Fig). In contrast, MLF is present at high levels in the nucleus of larval crystal cells (S4B Fig). Moreover, MLF expression in this lineage is not affected by *dnaj-1* loss (S4C and S4F Fig). We thus surmised that the presence of high levels of nuclear MLF might prevent Lz degradation in the absence of DnaJ-1.

To test this hypothesis, we designed two complementary experiments. On the one hand, we assessed whether MLF over-expression in Kc167 cells could protect Lz from degradation following *dnaj-1* knockdown. Lz level dropped when Kc167 cells were treated with a dsRNA targeting *dnaj-1* (Fig 4G) and increased upon over-expression of MLF (Fig 4F). Strikingly though, and in line with the observations in *dnaj-1* mutant crystal cells, the level of Lz was not reduced but further increased when *dnaj-1* was knocked down in MLF-overexpressing cells (Fig 4H and 4I). On the other hand, we asked whether Lz would still be stable in *dnaj-1* mutant crystal cells if MLF level is decreased. Accordingly, we expressed a dsRNA directed against *mlf* in lz>GFP^+^ cells, which caused a significant and similar knock-down of MLF in wild-type and *dnaj-1* mutant larvae (S4D–S4F Fig). Remarkably, we found that the drop in Lz protein level caused by *mlf* down-regulation was significantly enhanced in *dnaj-1* deficient larvae, while the *dnaj-1* mutation alone increased Lz level (Fig 4J–4N). Hence it appears that in the absence of DnaJ-1, high levels of MLF prevent Lz degradation.

Given that chaperones are important for proper protein folding [35, 36], we postulated that Lz proteins accumulating in crystal cells in the absence of DnaJ-1 might be less active. Thus increasing Lz expression might be sufficient to rescue lz>GFP^+^ cell number and size. In addition, although re-expressing Lz is sufficient to restore lz>GFP^+^ cell number in *mlf* mutant larvae [26], it is not known whether this also rescues lz>GFP^+^ cell size. Interestingly, lz>GFP^+^ cell count and cell size were restored to wild-type levels when we enforced Lz expression in this lineage either in *mlf* or *dnaj-1* mutant larvae (Fig 4O and 4P). We thus conclude that DnaJ-1 and MLF act together to control crystal cell development by regulating Lz activity *in vivo*

### MLF and DnaJ-1 control crystal cell differentiation

**Fig 5:**
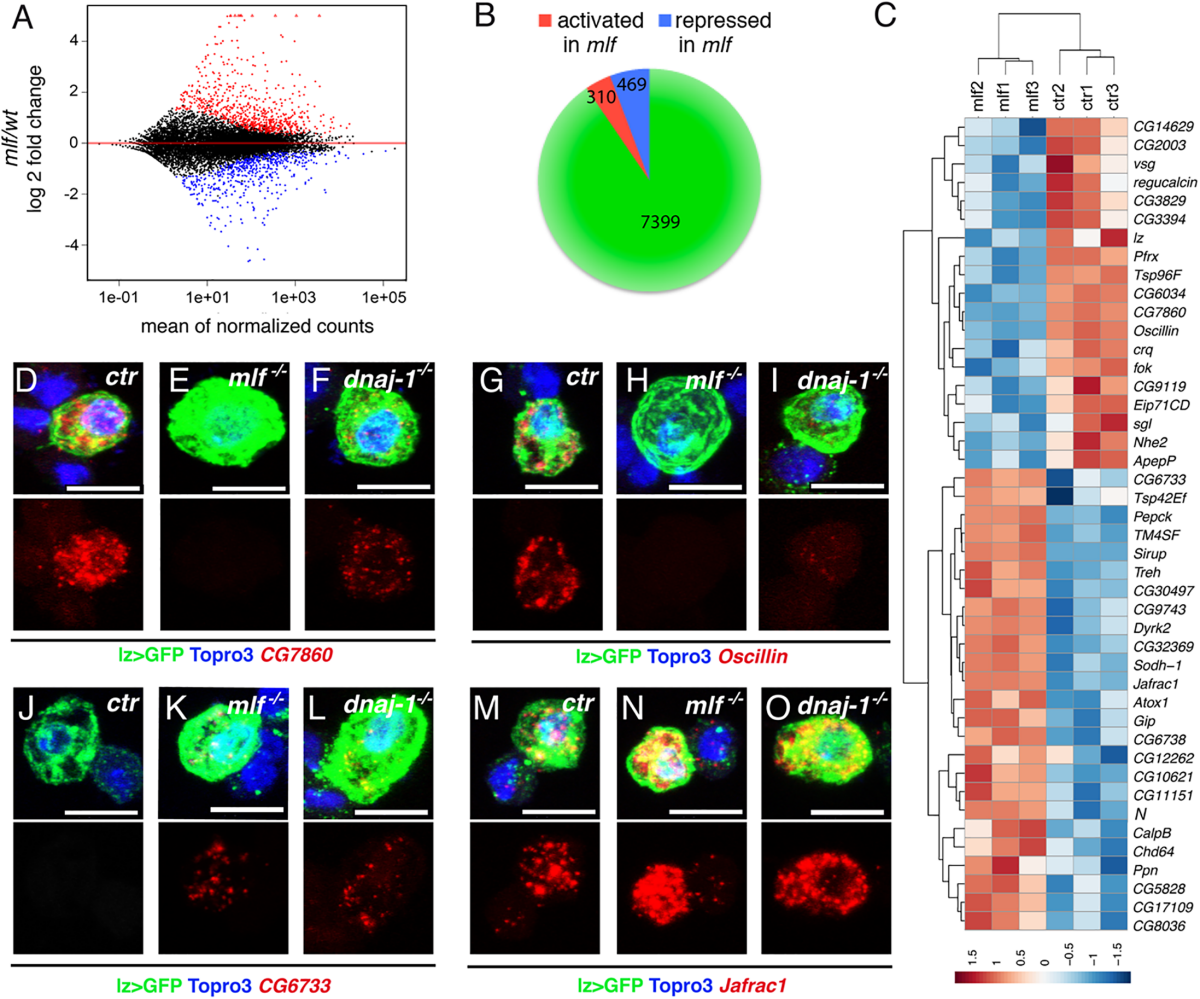
MLF and DnaJ-1 control crystal cell differentiation. (A) MA-plot of DESeq2 results for RNAseq data comparison between control and *mlf*^*-/-*^ lz>GFP^+^ blood cells sorted by FACS from third instar larvae. Genes that are significantly upregulated or downregulated in the *mlf* mutant (adjusted p-value<0.01) are highlighted in red or blue, respectively. Red triangles: genes with log_2_ fold change >5. (B) Pie chart showing the number of expressed genes in lz>GFP^+^ cells and the number of upregulated (red) or downregulated (blue) genes in the *mlf* mutant. (C) Heat map of “crystal cell”-associated genes differentially expressed (p-value<0.01) between control and *mlf* mutant lz>GFP^+^ cells. Differential gene expression as per comparison to the mean of the 6 samples (*ctr 1*, *2*, *3* and *mlf 1*, *2*, *3*) is displayed as log_2_ scale. Hierarchical clustering was performed using R-Bioconductor. (D-O) Immunostainings against GFP and *in situ* hybridization against *CG7860* (D-F), *Oscillin* (G-I), *Jafrac1* (J-L) and *CG6733* (M-O) in blood cells from *lz-GAL4*,*UAS-mCD8-GFP/+* control (D, G, J, M), *mlf*^*-/-*^ (E, H, K, N) or *dnaj-1*^*-/-*^ (F,I, L,O) third instar larvae. RNA expression only is shown in the lower panels. Nuclei were stained with Topro3. Scale bar: 10 μm.

In parallel, to gain further insights into the function of MLF in the control of crystal cell development, we established the transcriptome of circulating lz>GFP^+^ blood cells in wild-type and *mlf* larvae. Heterozygous *lz-GAL4*,*UAS-mCD8-GFP* L3 larvae carrying or lacking a *mlf* null mutation were bled, lz>GFP^+^ cells were collected by FACS and their gene expression profile was determined by RNA sequencing (RNAseq) from biological triplicates. Using *Drosophila* reference genome dm3, we detected the expression of 7399 genes (47% of the total fly genes) in each of the 6 samples (Fig 5B and S1 Table). Consistent with the role of the crystal cells as the main source of phenoloxidases [39], the two most strongly expressed genes were *PPO1* and *PPO2*. In addition, *lz* expression as well as that of several other crystal cell markers was readily detected (see below). It was recently shown that larval circulating Lz^+^ cells derive from plasmatocytes, which express Hemolectin (Hml) and Nimrod C1 (NimC1), and transdifferentiate into crystal cells [40]. Accordingly, we detected the expression of these genes, as well as other “plasmatocytes” markers such as *peroxidasin* and *croquemort* (which were actually shown to be also expressed in crystal cells [41, 42]) in lz>GFP^+^ cells.

Using DESeq2 to identify differentially expressed genes between wild-type and *mlf* mutant lz>GFP^+^ cells, we found 779 genes with significantly altered expression (adjusted p-value <0.01): the transcript level of 469 genes was decreased and that of 310 genes was increased in the absence of MLF (Fig 5A and 5B, and S2 Table). In line with our previous *in situ* hybridization results [26], RNAseq analysis did not reveal a significant modification of *PPO1* or *PPO2* expression in the absence of *mlf*. However, the *lz* transcript level was reduced by ±2 fold (p-value = 0.0018), which could be due to defective maintenance of the *lz* auto-activation loop [43]. To assess whether other crystal cell markers were affected by *mlf*, we established a compilation of genes expressed in (embryonic or larval) crystal cells based on Flybase data mining and re-examination of Berkeley *Drosophila* Genome Project *in situ* hybridizations (http://insitu.fruitfly.org/cgi-bin/ex/insitu.pl) (S3 Table). Among these 129 genes (*i*.*e*. excluding *mlf* itself), 44 (34%) were differentially expressed in the absence of *mlf* (19 repressed and 25 activated) (Fig 5C), indicating a strong over-representation of deregulated gene in the “crystal cell” gene set as compared to all expressed genes (p-value = 2.6x10^-13^, hypergeometric test) and showing that *mlf* plays a crucial role for proper crystal cell differentiation.

To substantiate these results, we analyzed by *in situ* hybridization the expression of 4 genes that were either down-regulated (*CG7860* and *Oscillin*) or up-regulated (*CG6733* and *Jafrac1*) in the *mlf* mutant. *CG7860* and *Oscillin* were specifically expressed in lz>GFP^+^ but not in the surrounding lz>GFP^-^ hemocytes in wild-type conditions (Fig 5D and 5G). Consistent with our RNAseq data, the expression of *CG7860* and *Oscillin* was strongly reduced in *mlf* mutant larvae. Although *CG6733* is expressed in embryonic crystal cells [43], we did not detect its expression in circulating hemocytes of wild-type larvae, but it was expressed in the lz>GFP^+^ lineage in *mlf* larvae (Fig 5J and 5K). Finally, *Jafrac1* expression increased in lz>GFP^+^ cells of *mlf* mutant larvae as compared to wild-type, whereas its (lower) expression in lz>GFP^-^ blood cells seemed similar (Fig 5M and 5N). These data thus confirm the RNAseq results and demonstrate that MLF controls the expression of several crystal cell markers. Since the above results indicate that MLF functionally interacts with DnaJ-1 during crystal cell development, we also tested whether these four genes were deregulated in the *dnaj-1* mutant. As for *mlf*, we observed that a *dnaj-1* mutation caused a down-regulation of *CG7860* and *Oscillin* and an up-regulation of *CG6733* and *Jafrac1* expression in lz>GFP^+^ blood cells (Fig 5F, 5I, 5L and 5O).

In sum it appears that the loss of *mlf* or *dnaj-1* leads to a deregulation of the crystal cell gene expression program characterized both by the overexpression and the downregulation of crystal cell markers. Therefore *mlf* and *dnaj-1* are required for proper differentiation of the Lz^+^ blood cell lineage.

### MLF and DnaJ-1 control Lz^+^ cell number and size by repressing Notch signaling

Interestingly, the levels of *Notch* receptor transcripts were significantly higher in the *mlf* mutant (p = 1.3x10^-6^) (Fig 5C). Notch signaling plays a key role in crystal cell development [27]: Notch is first activated by its ligand Serrate to specify Lz^+^ cells (crystal cell precursors) and its activation is subsequently maintained in Lz^+^ cells in a ligand-independent manner to promote crystal cell growth and survival [29–31, 40, 44]. The rise in lz>GFP^+^ cell number and size observed in *mlf* and *dnaj-1* mutant could thus be due to increased ligand-independent Notch signaling. However, the role of Notch signaling in crystal cell growth and survival has been mainly investigated in the larval lymph gland [30, 31]. In agreement with these investigations, inhibiting the Notch pathway in circulating Lz^+^ cells, either by down-regulating the expression of Suppressor of Hairless [Su(H)], the core transcription factor in the Notch pathway, or by overexpressing Suppressor of Deltex [Su(dx)], a negative regulator of Notch [45], resulted in a decrease in lz>GFP^+^ cell number and impaired their growth, whereas the overactivation of Notch signaling consecutive to the expression of a constitutively active Su(H)-VP16 fusion protein [46], caused a strong increase in lz>GFP^+^ cell number and size (S5 Fig).

**Fig 6:**
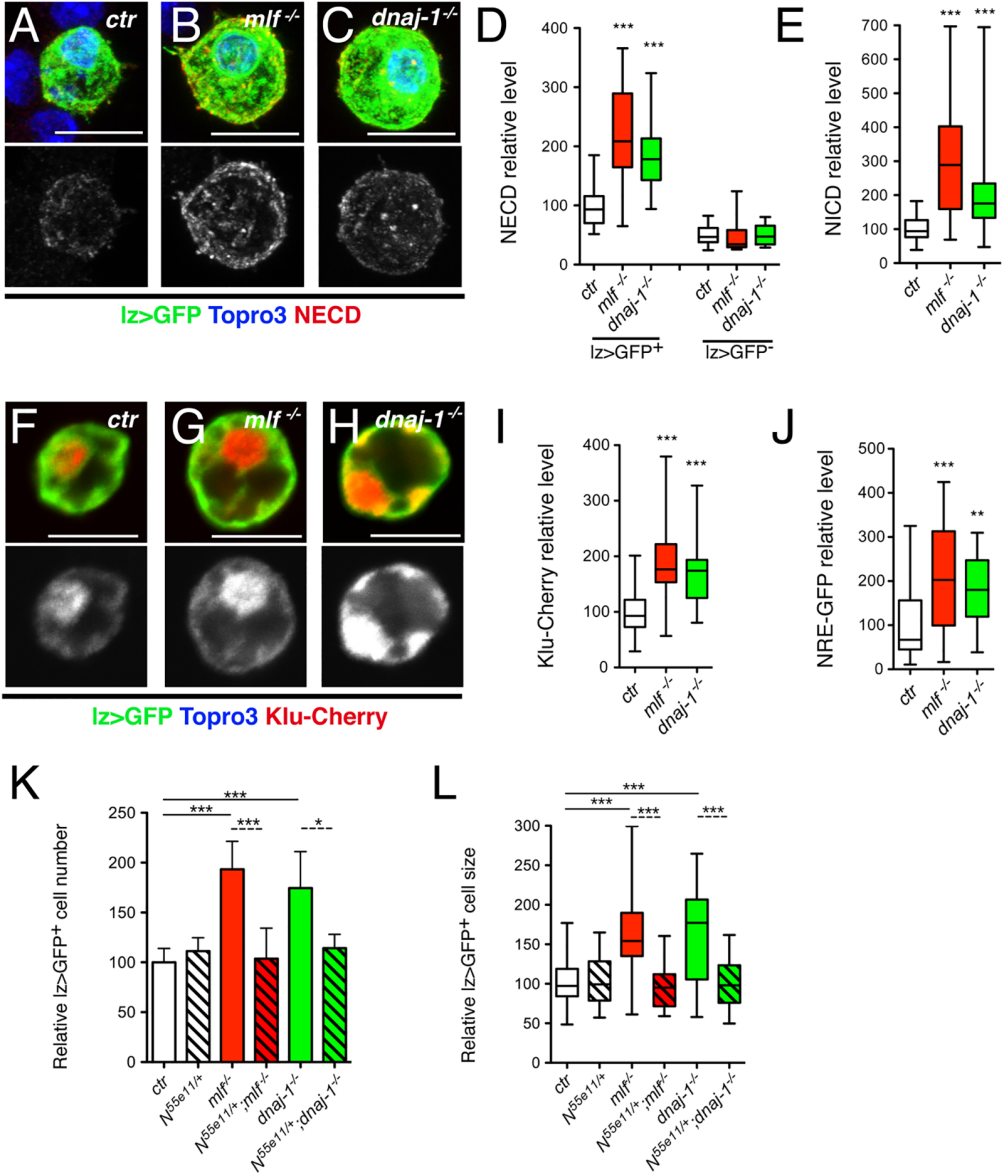
The increase in lz>GFP^+^ cell number and size in *mlf* and *dnaj-1* mutant larvae is caused by overactivation of the Notch signaling pathway. (A-C) Immunostainings against Notch (NECD: Notch extracellular domain) in blood cells from *lz-GAL4*,*UAS-mCD8-GFP/+* control (A), *mlf*^*-/-*^ (B) and *dnaj-1*^*-/-*^ (C) larvae. The immunostaining against Notch protein only is shown in the lower panels. Nuclei were stained with Topro3. (D) Quantification of NECD immunostainings in lz>GFP^+^ and lz>GFP^-^ blood cells from control, *mlf*^*-/-*^ and *dnaj-1*^*-/-*^ larvae. (E) Quantification of NICD (Notch intracellular domain) immunostainings in lz>GFP^+^ blood cells from control, *mlf*^*-/-*^ and *dnaj-1*^*-/-*^ larvae. (F-H) Expression of the Notch pathway reporter Klu-Cherry in lz>GFP^+^ blood cells from control, *mlf*^*-/-*^ or *dnaj-1*^*-/-*^ larvae. Klu-Cherry expression only is shown in the lower panels. (I) Corresponding quantification of Klu-Cherry level. (J) Quantification of the expression level of the Notch pathway reporter NRE-GFP in PPO1-expressing cells from control, *mlf*^*-/-*^ or *dnaj-1*^*-/-*^ larvae. (K, L) Relative lz>GFP^+^ blood cell number (K) and size (L) in third instar larvae of the indicated genotypes.

Then we further investigated the level of Notch expression and activation in *mlf* and *dnaj-1* mutant blood cells. Immunostaining using an antibody against the Notch extracellular domain (NECD) showed that Notch accumulated at higher levels in lz>GFP^+^ cells of *mlf* and *dnaj-1* mutant larvae than in wild-type conditions (Fig 6A–6C). Quantitative analyses confirmed that *mlf* loss caused a significant increase in Notch level in lz>GFP^+^ cell, whereas the (lower) expression of Notch in lz>GFP^-^ blood cells was not affected (Fig 6D). Similar results were obtained when we measured Notch protein levels using an antibody directed against its intra-cellular domain (NICD) (Fig 6E and S6 Fig). Thus Notch level is specifically increased in lz>GFP^+^ cells of *mlf* and *dnaj-1* mutants. Next, we tested whether this resulted in increased signaling by monitoring the expression of two Notch signaling pathway reporter genes expressed in larval crystal cells: Klumpfuss-Cherry [31] and NRE-GFP [47]. Both *mlf* and *dnaj-1* loss were associated with a strong increase in the expression of these reporters (Fig 6F–6J). Thus *mlf* and *dnaj-1* are required to tune down Notch signaling in the crystal cell lineage. Finally, we asked whether the rise in lz>GFP^+^ cell size and/or number observed in *mlf* and *dnaj-1* mutants depends on Notch. Strikingly, when we reduced *Notch* dosage by introducing one copy of the *N*^*55e11*^ null allele in these mutants, both parameters were restored to control levels, while *N*^*55e1*1^ heterozygote mutation had no effect *per se* (Fig 6K and 6L). Collectively, these data strongly support the hypothesis that the increase in Notch level underlies lz>GFP^+^ cell expansion in *mlf* and *dnaj-1* mutants.

### MLF and DnaJ-1 are required to turn-down Notch expression during crystal cell maturation

**Fig 7:**
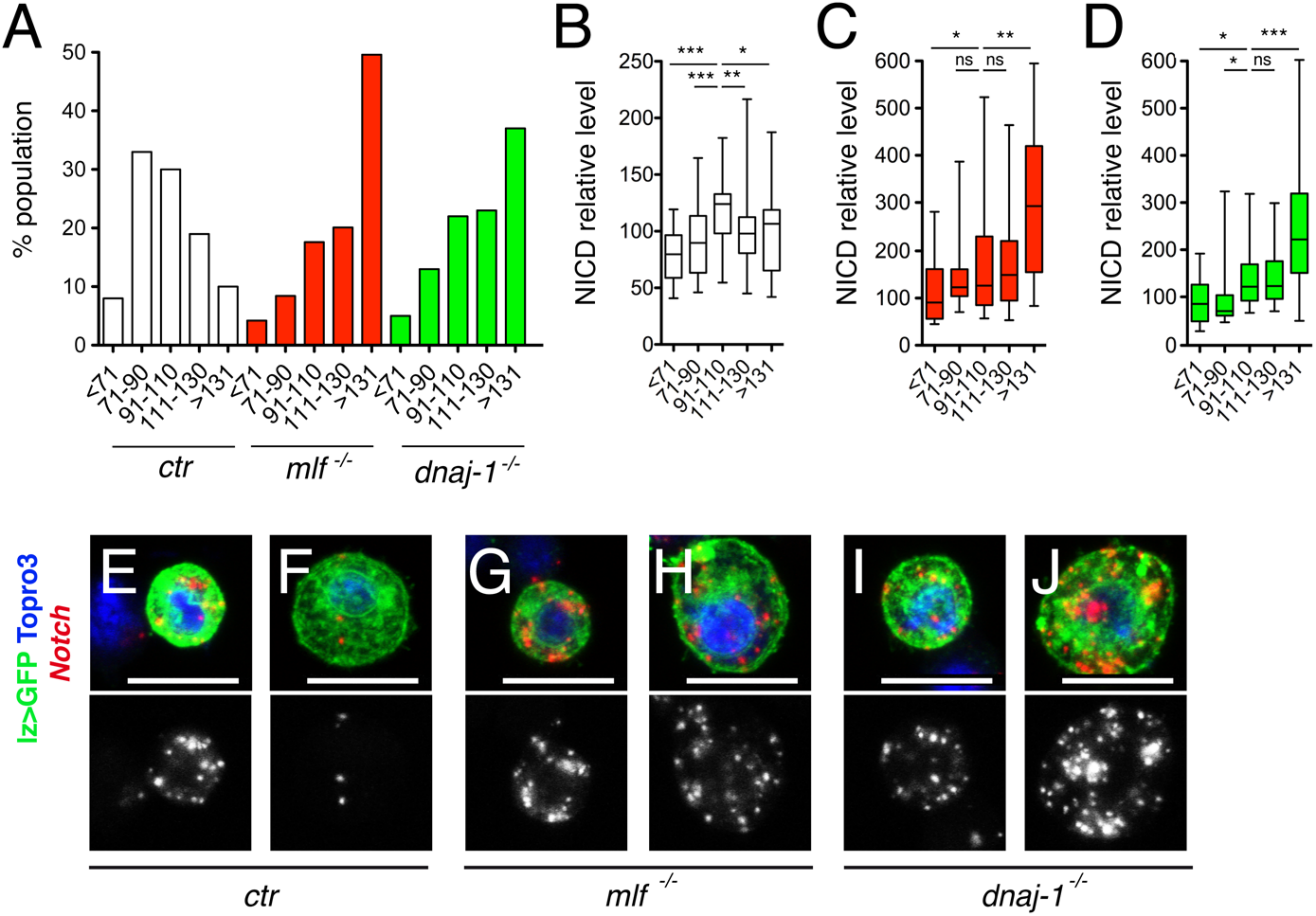
MLF and DnaJ-1 are required to turn down Notch expression in large crystal cells. (A) Quantificationd of the proportion of lz>GFP^+^ cells according to their size in control, *mlf*^*-/-*^ or *dnaj-1*^*-/-*^ larvae. Cells were grouped into 5 categories as compared to the mean size of lz>GFP^+^ cells in the control condition. (B-D) Quantification of NICD immunostaining (relative to control) in each of the five lz>GFP^+^ cell size categories in control (B), *mlf*^*-/-*^ (C) and *dnaj-1*^*-/-*^ (D) larvae. *:p-value<0.05, **: p-value<0.01, *** p-value<0.001, n.s.: not significant. (E-J) Fluorescent immunostainings of GFP and *in situ* hybridizations of *Notch* in circulating blood cells from *lz-GAL4*,*UAS-mCD8-GFP/+* third instar larvae of the indicated genotypes. Representative images of *Notch* expression in small/medium (E, G, I) *versus* large (F, H, J) lz>GFP^+^ cells. Scale bar: 10 μm. Nuclei were stained with Topro3. The lower panels show *Notch* expression only.

It was shown that crystal cells tend to increase their size as they mature in response to Notch signaling [31, 40], which is consistent with the results we obtained by manipulating Notch signaling activity in Lz^+^ cells (S5 Fig). To better characterize the defects associated with *mlf* or *dnaj-1* loss, we analyzed the distribution of lz>GFP^+^ cells as well as Notch level according to lz>GFP^+^ cell size categories. Whereas cells more than 1.3-fold larger than the mean wild-type cell size represented a small fraction (±10%) of the lz>GFP^+^ population in wild-type larvae, they constituted the prevalent population in *mlf* or *dnaj-1* mutant (respectively 49.6 and 37%) (Fig 7A). Interestingly, Notch protein level was maximum in the population of lz>GFP^+^ cells of mean cell size but lower in larger cells of wild-type larvae (Fig 7B), whereas it continued to increase in the larger cell populations of *mlf* or *dnaj-1* larvae (Fig 7B–7D). Actually we observed a similar trend when we monitored *Notch* expression by *in situ* hybridization. In wild-type larvae, *Notch* transcripts were readily seen in small/medium lz>GFP^+^ cells but barely detectable in large lz>GFP^+^ cells (Fig 7E and 7F). In contrast, *Notch* transcripts continued to accumulate in large lz>GFP^+^ cells from *mlf* or *dnaj-1* mutant larvae (Fig 7H and 7J). Hence, MLF/DnaJ-1 loss is associated with the accumulation of large crystal cells exhibiting aberrant maintenance of *Notch* expression. Since the Notch pathway is activated in a ligand-independent manner in Lz^+^ cells [30], a tight regulation of the level of Notch is particularly critical to control crystal cell growth and number. All together, our data suggest that in *mlf* or *dnaj-1* mutant larvae, *Notch* expression fails to be turned down when lz>GFP^+^ cells reach a critical size, leading to the maintenance of a high level of Notch signaling and thus to increased crystal cell growth and survival.

### High levels of Lz prevent accumulation of lz>GFP^+^ cells and repress Notch expression/signaling

**Fig 8:**
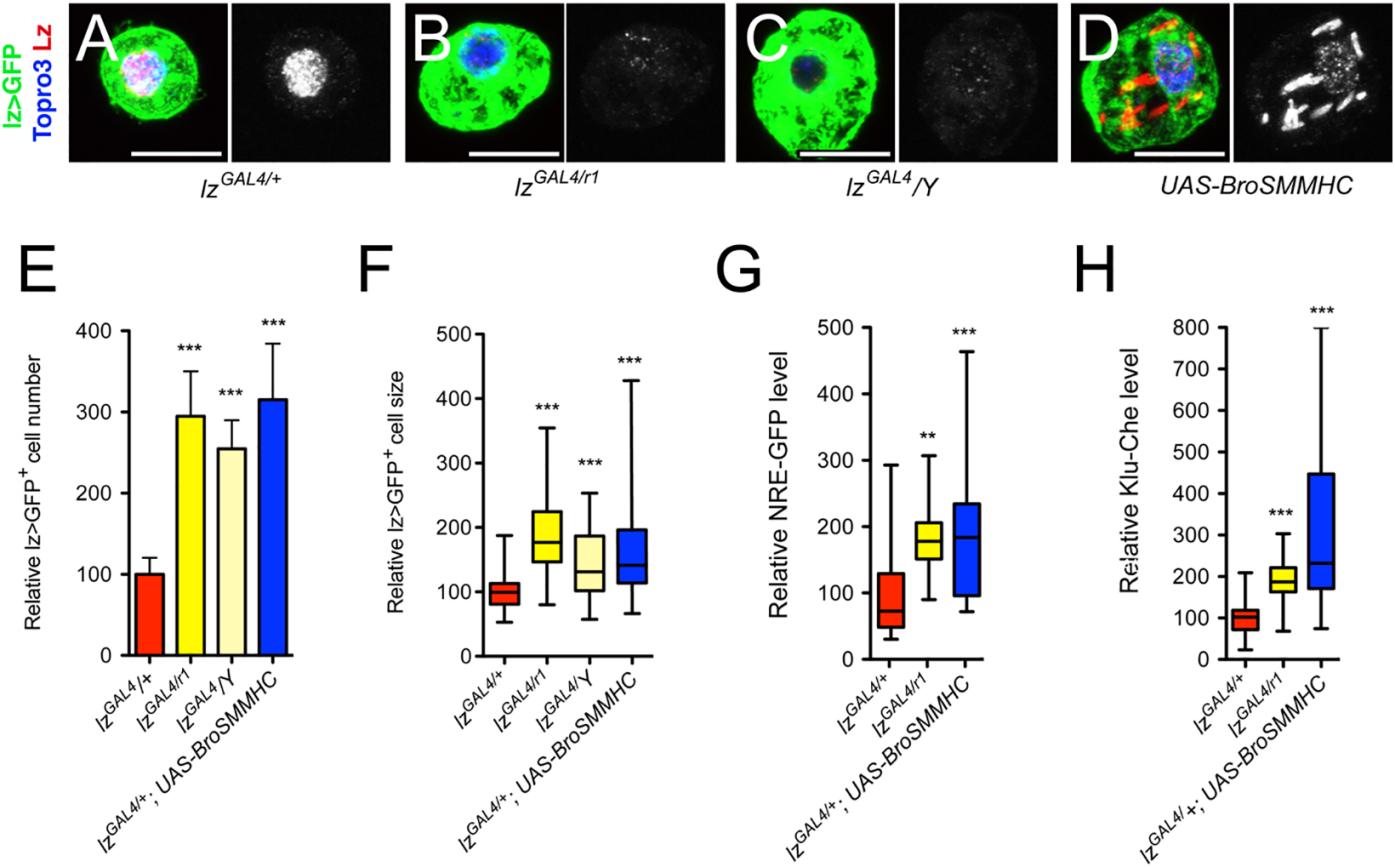
High levels of Lz prevent lz>GFP^+^ cell accumulation and Notch signaling overactivation. (A-D) Fluorescent immunostainings of Lz in circulating blood cells from *lz-GAL4*, *UAS-mCD8-GFP/+* (A, control), *lz-GAL4*, *UAS-mCD8-GFP/lz*^*r1*^ (B), *lz-GAL4*, *UAS-mCD8-GFP/Y* (C) and *lz-GAL4*, *UAS-mCD8-GFP/+; UAS-BroSMMHC* (D) third instar larvae. Nuclei were stained with Topro3. Scale bar: 10μm. Lz immunostaining only is shown in the right panels. (E-H) Quantifications of lz>GFP^+^ cell number (E) and size (F) as well as NRE-GFP (G) and Klu-Cherry (H) expression levels in third instar larvae of the indicated genotypes. **: p-value<0.01, *** p-value<0.001.

We showed above that forcing the expression of Lz rescues the increase in crystal cell number and size caused by *mlf* or *dnaj-1* loss. It is thus plausible that this RUNX transcription factor directly participates in down-regulation of Notch signaling. To explore this hypothesis, we asked whether a reduction in *lz* activity might cause an expansion of the Lz^+^ cell lineage associated with an over-activation of the Notch pathway. Accordingly, we introduced the *lz*^*r1*^ null allele into the *lz*^*GAL4*^ context. This hypomorphic allelic combination caused a decrease in Lz expression (Fig 8B) and resulted in an increase in lz>GFP^+^ cell number and size (Fig 8E and 8F). Interestingly, *lz*^*GAL4*^*/Y* hemizygous larvae displayed similar phenotypes (Fig 8C, 8E and 8F), indicating that this P{GAL4} insertion in *lz* alters its expression in the crystal cell lineage. As an alternate strategy, we interfered with Lz activity by expressing a fusion protein between Lz partner Brother (*Drosophila* CBFß homolog) and the non-muscular myosin heavy chain SMMHC [48]. This chimera mimics the CBFß-MYH11 fusion protein generated by the Inv(16) translocation in human AML and can sequester RUNX factors in the cytoplasm [1, 49]. Bro-SMMHC expression in lz>GFP^+^ cells titrated Lz from the nucleus and also caused an increase in lz>GFP^+^ cell number and size (Fig 8D–8F). Importantly, the expression of the Notch pathway reporters NRE-GFP and Klu-Cherry was strongly increased in *lz*^*GAL4/*^*lz*^*R1*^ mutant or upon Bro-SMHCC expression in the Lz^+^ blood cell lineage (Fig 8G and 8H). Moreover, knocking down Su(H) or over-expressing the Notch protein inhibitor Su(dx) was sufficient to prevent the rise in lz>GFP^+^ cell number and size of *lz*^*GAL4*^*/Y* hemyzigotes (S5 Fig). Thus, a reduction in *lz* activity causes similar defects as the *mlf* or *dnaj-1* mutations and likely involves the overactivation of the Notch pathway.

**Fig 9:**
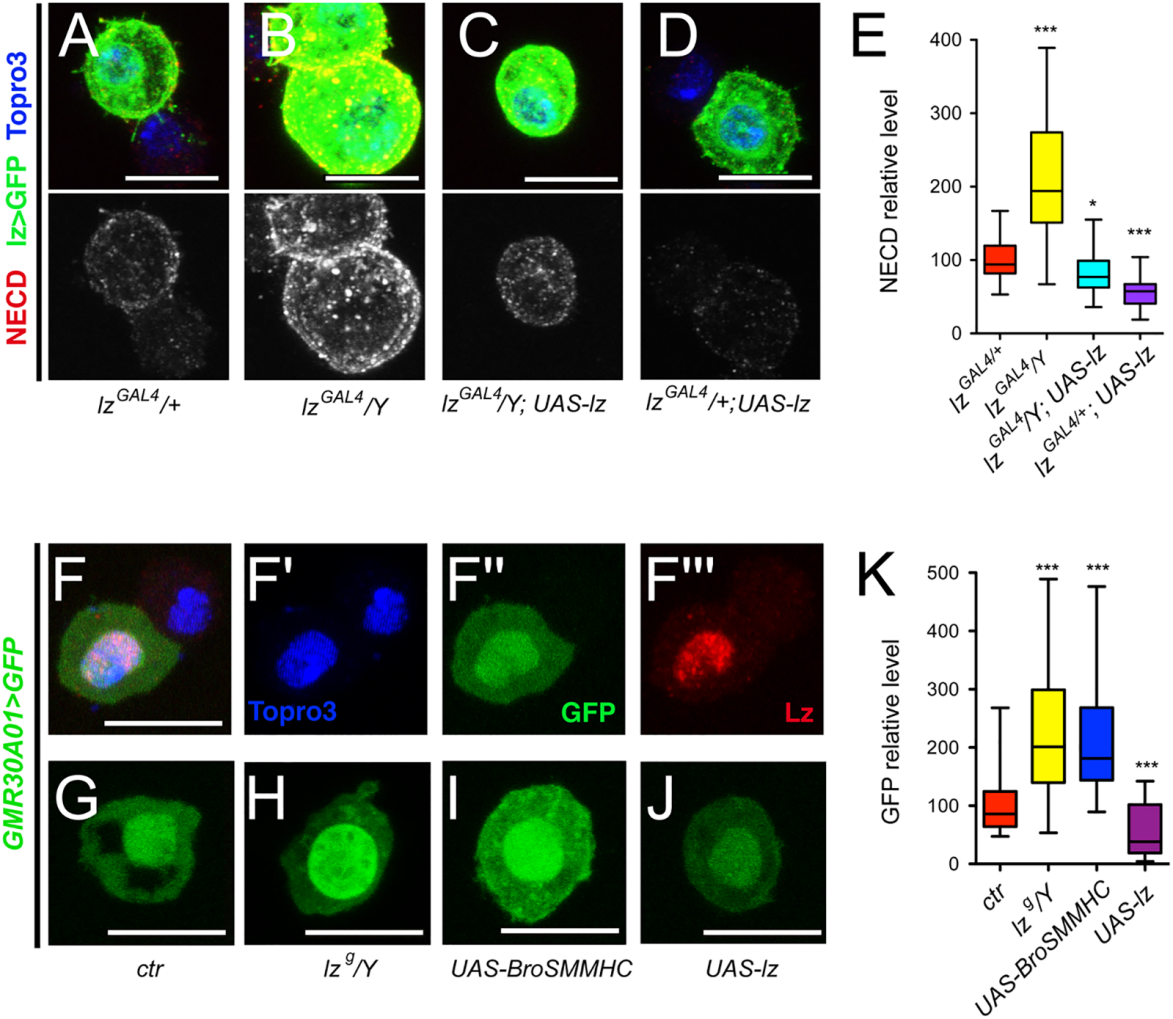
Lz represses *Notch* expression. (A-D) Immunostainings against NECD (Notch extracellular domain) in blood cells from *lz-GAL4*, *UAS-mCD8-GFP/+* (A), *lz-GAL4*, *UAS-mCD8-GFP/Y* (B), *lz-GAL4*, *UAS-mCD8-GFP/Y; UAS-lz* (C) and *lz-GAL4*, *UAS-mCD8-GFP/+; UAS-lz* (D) third instar larvae. NECD immunostaining only is shown in the lower panels. Nuclei were stained with Topro3. (E) Corresponding quantifications of NECD in lz>GFP^+^ blood cells. (F-F”‘) Immunostaining against Lz in circulating blood cells from *Notch*^*GMR30A01*^*-GAL4*, *UAS-GFPnls* third instar larvae. Nuclei were stained with Topro3. (F’-F”‘): single channel images. (G-J) *Notch*^*GMR30A01*^*-GAL4*-driven expression of GFP in circulating blood cells from larvae of the indicated genotypes. (K) Corresponding quantifications of the level of GFP. (A-D, F-J) Scale bar: 10μm. (E, K) *: p-value<0.05, *** p-value<0.001.

Then we analyzed the relathionship between Lz and Notch levels. In Lz^+^ cells of increasing size, Lz levels continuously increased while Notch became less abundant (S7A Fig). This suggested that Lz level rises as crystal cells grow/mature and, in view of the above results, we surmised that this increase might participate in the down-regulation of the Notch receptor. Indeed, we found that the Notch receptor level was significantly augmented in lz>GFP^+^ cells of hypomorphic *lz*^*GAL4*^*/Y* hemyzigote mutant larvae, whereas it was reduced when Lz was over-expressed (Fig 9A–9E). In addition, the increase in Notch expression observed in *lz*^*GAL4*^*/Y* larvae was suppressed by forcing Lz expression. Moreover, *in situ* hybridization experiments revealed that, unlike in control larvae, *Notch* expression was not repressed in large lz>GFP^+^ cells in *lz*^*GAL4*^*/Y* larvae (S7 Fig). Therefore *Notch* might be a direct transcriptional target of Lz. By analyzing the expression of different GAL4 lines that cover potential *Notch* regulatory regions [50], we identified two lines that drive expression in circulating Lz^+^ blood cells (Fig 9F and S7 Fig). The regulatory elements carried by these two lines (GMR30A01 and GMR30C06) overlap on a 668bp DNA segment that contains two consensus binding sites for RUNX transcription factors conserved in other *Drosophila* species (S7A Fig), suggesting that Lz might directly regulate *Notch* transcription by targeting this region. We thus tested the effect of Lz dosage manipulation on the activity of this enhancer-GAL4 line. Strikingly, a hypomorphic *lozenge* mutation (*lz*^*g*^*/Y*) [51] or the expression of Bro-SMMHC caused an increase in the expression of this enhancer, whereas the over-expression of Lz resulted in its down-regulation (Fig 9G–9K). These findings strongly argue that Lz directly represses *Notch* expression.

All together, these results demonstrate that high levels of Lz are required to prevent the accumulation of over-grown lz>GFP^+^ cells as well as over-activation of the Notch pathway, and we propose that Lz-mediated repression of *Notch* transcription is critical during this process.

## Discussion

Members of the RUNX and MLF families have been implicated in the control of blood cell development in mammals and *Drosophila* and deregulation of their expression is associated with human hemopathies including leukemia [1, 9, 15, 52]. Our results establish the first link between the MLF/DnaJ-1 complex and the regulation of a RUNX transcription factor *in vivo*. In addition, our data show that the stabilization of Lz by the MLF/DnaJ-1 complex is critical to control Notch expression and signaling and thereby blood cell growth and survival. These findings pinpoint the specific function of the Hsp40 chaperone DnaJ-1 in hematopoiesis, reveal a potentially conserved mechanism of regulation of RUNX activity and highlight a new layer of control of Notch signaling at the transcriptional level.

In line with results published as this manuscript was in preparation [20], we found that MLF binds DnaJ-1 and Hsc70-4 and that these two proteins, like MLF, are required for Lz stable expression in Kc167 cells. In addition, our data show that MLF and DnaJ-1 bind to each other *via* evolutionarily conserved domains and also interact with Lz, suggesting that Lz is a direct target of a chaperone complex formed by MLF, DnaJ-1 and Hsc70-4. Of note, a systematic characterization of Hsp70 chaperone complexes in human cells identified MLF1 and MLF2 as potential partners of DnaJ-1 homologs, DNAJB1, B4 and B6 [53], a finding corroborated by Dyer *et al*. [20]. Therefore, the MLF/DnaJ-1/Hsc70 complex could play a conserved role in mammals, notably in the regulation of the stability of RUNX transcription factors. How MLF acts within this chaperone complex remains to be determined. *In vivo*, we demonstrate that *dnaj-1* mutations lead to defects in crystal cell development strikingly similar to those observed in *mlf* mutant larvae and we show that these two genes act together to control Lz^+^ cells development by impinging on Lz activity. Our data suggest that in the absence of DnaJ-1, high levels of MLF lead to the accumulation of defective Lz protein whereas lower levels of MLF allow its degradation. We thus propose that MLF stabilizes Lz and, together with DnaJ-1, promotes its proper folding/conformation. In humans, DnaJB4 stabilizes wild-type E-cadherin but induces the degradation of mutant E-cadherin variants associated with hereditary diffuse gastric cancer [54]. Thus the fate of DnaJ client proteins is controlled at different levels and MLF might be an important regulator in this process.

In this work, we present the first null mutant for a gene of the *DnaJB* family in metazoans and our results demonstrate that a DnaJ protein is required *in vivo* to control hematopoiesis. There are 16 DnaJB and in total 49 DnaJ encoding genes in mammals and the expansion of this family has likely played an important role in the diversification of their functions [55, 56]. DnaJB9 overexpression was found to increase hematopoietic stem cell repopulation capacity [57] and Hsp70 inhibitors have anti-leukemic activity [58], but the participation of other DnaJ proteins in hematopoiesis or leukemia has not been explored. Actually DnaJ’s molecular mechanism of action has been fairly well studied but we have limited insights as to their role *in vivo*. Interestingly though, both DnaJ-1 and MLF suppress polyglutamine protein aggregation and cytotoxicity in *Drosophila* models of neurodegenerative diseases [17, 23, 24, 59–63, 64], and this function is conserved in mammals [24, 25, 65, 66]. It is tempting to speculate that MLF and DnaJB proteins act together in this process as well as in leukemogenesis. Thus a better characterization of their mechanism of action may help develop new therapeutic approaches for these diseases.

As shown here, *mlf* or *dnaj-1* mutant larvae harbor more crystal cells than wild-type larvae. This rise in Lz^+^ cell number is not due to an increased induction of crystal cell fate as we could rescue this defect by re-expressing DnaJ-1 or Lz with the *lz-GAL4* driver, which turns on after crystal cell induction, and it was also observed in *lz* hypomorph mutants, which again suggests a post-*lz* / cell fate choice process. Moreover *mlf* or *dnaj-1* mutant larvae display a higher fraction of the largest lz>GFP^+^ cell population, which could correspond to the more mature crystal cells [31, 40]. It is thus tempting to speculate that *mlf* or *dnaj-1* loss promotes the survival of fully differentiated crystal cells. Our RNAseq data demonstrate that *mlf* is critical for expression of crystal cell associated genes, but we observed both up-regulation and down-regulation of crystal cell differentiation markers in *mlf* or *dnaj-1* mutant Lz^+^ cells. Also these changes did not appear to correlate with crystal cell maturation status since we found alterations in gene expression in the mutants both in small and large Lz^+^ cells. In addition our transcriptome did not reveal a particular bias toward decreased expression for “plasmatocyte” markers in Lz^+^ cells from *mlf*^*-*^ mutant larvae. Thus, it appears that MLF and DnaJ-1 loss leads to the accumulation of mis-differentiated crystal cells.

**Fig 10:**
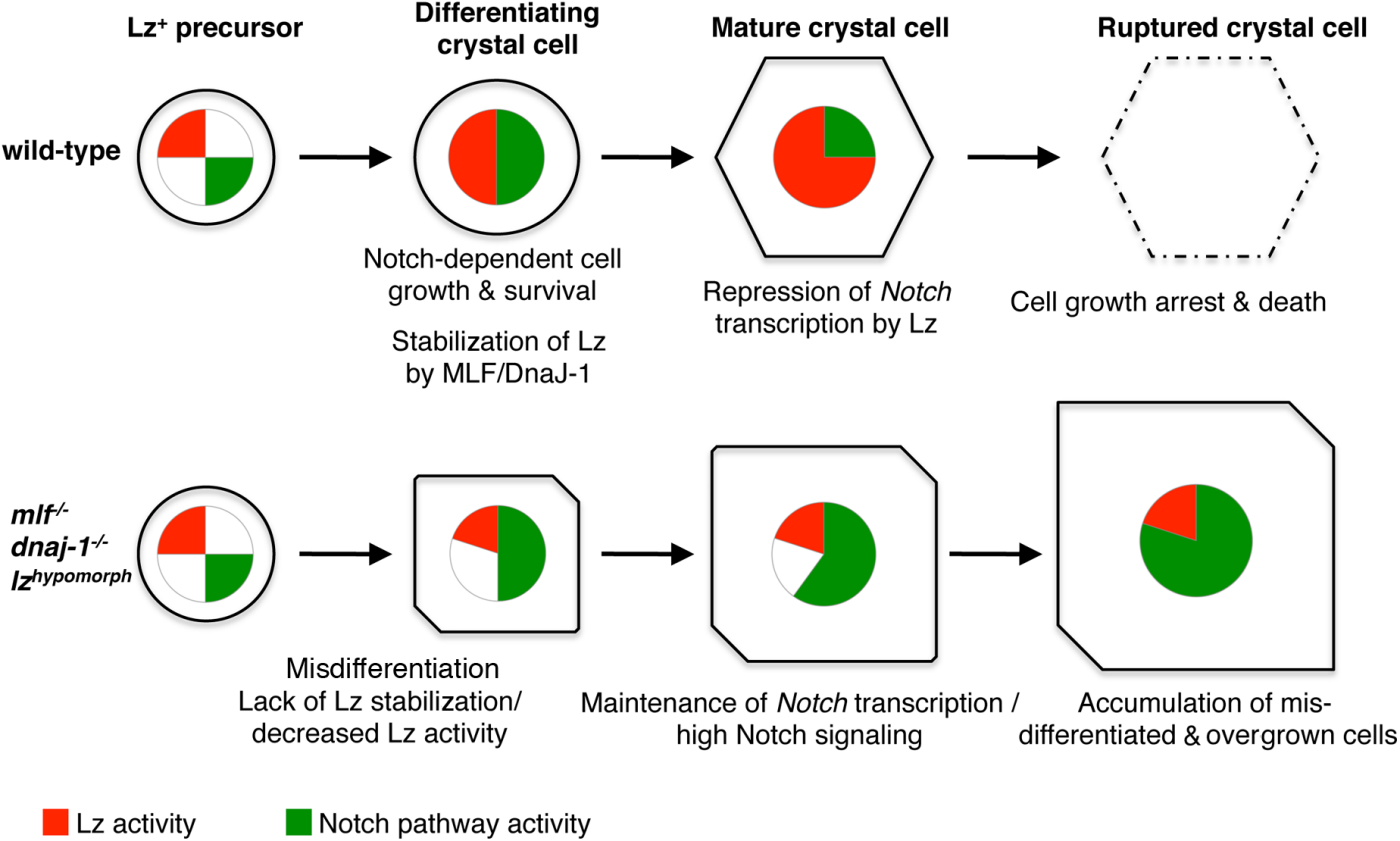
A model for the control of crystal cell development by MLF/DnaJ-1, Lz, and Notch. In wild-type conditions, *lz* expression is induced in crystal cell precursors and Lz protein gradually accumulates thanks to its interaction with MLF/DnaJ-1. At the same time, ligand-independent Notch signaling promotes crystal cell growth and survival. Once it reaches a sufficient level, Lz represses *Notch* transcription. This leads to a down-regulation of Notch signaling, thereby limiting crystal cell growth and promoting the death (rupture) of mature crystal cells. In conditions where Lz activity is impaired (decreased expression or lack of stabilization by MLF/DnaJ-1), crystal cells do not differentiate properly and Notch activity is maintained at high levels, which causes the accumulation of a higher number of Lz^+^ cells and their overgrowth.

Our data support a model whereby MLF and DnaJ-1 act together to promote Lz accumulation, which in turn represses Notch transcription and signaling pathway to control crystal cell size and number (Fig 10). Indeed, we observe an abnormal maintenance of *Notch* expression in the larger Lz^+^ cells as well as an over-activation of the Notch pathway in the crystal cell lineage of *mlf* and *dnaj-1* mutants or when we interfere with Lz activity. Moreover our data as well as previously published experiments show that Notch activation promotes crystal cell growth and survival [30, 31, 40]. Importantly too the increase in Lz^+^ cell number and size observed in *mlf* or *dnaJ-1* mutant is suppressed when *Notch* dosage is decreased. Yet, some of the mis-differentiation phenotypes in the *mlf* or *dnaj-1* mutants might be independent of Notch since changes in crystal cell markers expression seem to appear before alterations in Notch are apparent. At the molecular level, our results suggest that Lz directly represses *Notch* transcription as we identified a Lz-responsive *Notch cis*-regulatory element that contains conserved RUNX binding sites. The activation of the Notch pathway in circulating Lz^+^ cells is ligand-independent and mediated through stabilization of the Notch receptor in endocytic vesicles [30, 45]. Hence a tight control of *Notch* expression is of particular importance to keep in check the Notch pathway and prevent the abnormal development of the Lz^+^ blood cell lineage. Notably, *Notch* transcription was shown to be directly activated by Notch signaling [67]. Such an auto-activation loop might rapidly go awry in a context in which Notch pathway activation is independent of ligand binding. By promoting the accumulation of Lz during crystal cell maturation, MLF and DnaJ-1 thus provide an effective cell-autonomous mechanism to inhibit Notch signaling. Further experiments will now be required to establish how Lz represses *Notch* transcription. RUNX factors can act as transcriptional repressors by recruiting co-repressor such as members of the Groucho family [68]. Whether MLF and DnaJ-1 directly contribute to Lz-induced-repression in addition to regulating its stability is an open question. MLF and DnaJ-1 were recently found to bind and regulate a common set of genes in cell culture [20]. They may thus provide a favorable chromatin environment for Lz binding or be recruited with Lz and/or favor a conformation change in Lz that allows its interaction with co-repressors. The scarcity of lz>GFP^+^ cells precludes a biochemical characterization of Lz, MLF and DnaJ-1 mode of action notably at the chromatin level but further genetic studies should help decipher their mode of action. While the post-translational control of Notch has been extensively studied, its transcriptional regulation seems largely overlooked [69]. Our findings indicate that this is nonetheless an alternative entry point to control the activity of this pathway. Given the importance of RUNX transcription factor and Notch signaling in hematopoiesis and blood cell malignancies [1, 2], it will be of particular interest to further study whether RUNX factors can regulate Notch expression and signaling during these processes in mammals.

In conclusion, our study shows that MLF and DnaJ-1 act together to regulate RUNX transcription factor activity, which in turn controls Notch signaling during hematopoiesis *in vivo*. We anticipate that the extraordinary genetic toolbox available in *Drosophila* will help shed further light on the mechanism of action of these evolutionarily conserved proteins and will bring valuable insights into the control of protein homeostasis by MLF and DnaJ-1 during normal or pathological situations.

## Materials and methods

### Fly strains

The following *Drosophila melanogaster* lines were used: *mlf*^*ΔC1*^, *UAS-mlf* [17], *UAS-ds-mlf* (National Institute of Genetics), *UAS-lz*, *lz*^*GAL4*^,*UAS-mCD8-GFP*, *lz*^*g*^, *lz*^*r1*^, *N*^*55e11*^, *UAS-dsSu(H)*, P*{EPgy2}DnaJ-1EY04359*, *UAS-dnaj-1*, *Def(3L)BSC884*, *vas-Cas9*, *UAS-GFPnls*, *NRE-GFP*, *GMR30C06*, *GMR30A01*, *UAS-dsSu(H)* (Bloomington *Drosophila* Stock Center), *Bc-GFP* [70], *Klu-mCherry* [31] *UAS-Bro-SMMHC* [48], *UAS-DnaJ-1ΔJ* [61], *UAS-dsSu(H)*, *UAS-Su(H)-VP16* [46], *UAS-Su(dx)* [71]. To generate *dnaj-1* deficient flies, we designed two guide RNA targeting *dnaj-1* locus (S4 Fig) and the corresponding DNA oligonucleotides (g2: GTCGACCACAACGCGCCGGATCAA; g3: GTCGCATCACAGTCACGCTTTCCT) were cloned in pCFD3 (Addgene). *vas-cas9* females were crossed to P*{EPgy2}DnaJ-1EY04359* males and the resulting embryos were injected using standard procedures with both pCFD3-g2 and pCFD3-g3 plasmids (500ng/ul). Deletion of the P{EPgy2}^EY04359^ transposon, as revealed by loss of the *w*^*+*^ marker, was screened for at the F2 generation, and deletion of *dnaj-1* locus was assessed by PCR and sequencing.

All crosses were conducted at 25°C on standard food medium as described in [72].

### Immunostainings and *in situ* hybridizations

For each sample, four third instar larvae were bled (or 5.10^3^ Kc167 cells were dispensed) in 1ml of PBS in 24-well-plate containing a glass coverslip. Unless mentioned otherwise, only female larvae were used. The hemocytes were centrifuged for 2 min at 900g, fixed for 20 min with 4% paraformaldehyde in PBS and washed twice in PBS. For immunostainings: cells were permeabilized in PBS-0.3% Triton (PBST) and blocked in PBST- 1% Bovine Serum Albumin (BSA). The cells were incubated with primary antibodies at 4°C over night in PBST-BSA, washed in PBST, incubated for 2h at room temperature with corresponding Alexa Fluor-labeled secondary antibodies (Molecular Probes), washed in PBST and mounted in Vectashield medium (Eurobio-Vector) following incubation with Topro3 (ThermoFisher). The following antibodies were used: anti-Lz, anti-Notch intracellular domain, anti-Notch extracellular domain (Developmental Studies Hybridoma Bank, DSHB), anti-MLF [73], anti-PPO1 [74], anti-GFP (Fisher Scientific), anti-HA (Sigma).

For *in situ* hybridizations: after fixation, the cells were washed and permeabilized in PBS- 0.1% Tween 20 (PBSTw), pre-incubated for 1h at 65°C in HB buffer (50% formamide, 2x SSC, 1 mg/ml Torula RNA, 0.05 mg/ml Heparin, 2% Roche blocking reagent, 0.1% CHAPS, 5 mM EDTA, 0.1% Tween 20) and incubated over-night with anti-sense DIG-labeled RNA probes (against *CG6733*, *CG7860*, *Jafrac*, *Notch* and *Oscillin)* diluted in HB. The samples were washed in HB for 1h at 65°C, in 50% HB- 50% PBSTw for 30 min at 65°C and three times in PBSTw for 20 min at room temperature. Then the cells were incubated for 30 min in PBSTw- 1% BSA before being incubated with anti-DIG antibody conjugated to alkaline phosphatase (Roche, 1/2000 in PBSTw) for 3h. After 4 washes in PBSTw, *in situ* hybridization signal was revealed with FastRed (Roche). The cells were then processed for immunostaining against GFP as described above, incubated in Topro3, washed in PBS and mounted in Vectashield medium for analysis.

Experiments were performed using at least biological triplicates. Samples were imaged with laser scanning confocal microscopes (Leica) and images were analyzed with ImageJ. Cell size and protein expression levels were measured on maximal intensity projections of Z-sections through the whole cell on a minimum of 25 cells per genotype.

### Plasmids

The following previously described plasmids were used: pAc-Lz-V5, 4xPPO2-Firefly luciferase (originally named 4xPO45-Fluc, [37]), pAc-MLF [17]. We generated the following *Drosophila* expression plasmids for C-terminally tagged or N-terminally tagged proteins using standard cloning techniques: pAc-Lz-EGFP, pAc-MLF-EGFP, pMT-MLF-V5-His, pAc-DnaJ-J1-EGFP, pAc-Hsc70-4-EGFP, pAc-3xHA-DnaJ-1 (2–334), pAc-3xHA-DnaJ-1 (P32S), pAc-3xHA-DnaJ-1 (58–334), pAc-3xHA-DnaJ-1 (2–156), pAc-3xHA-DnaJ-1 (2–191), pAc-3xHA-DnaJ-1 (2–269), pAc-3xHA-DnaJ-1 (157–334), pAc-3xHA-MLF (2–309), pAc-3xHA-MLF (2–147), pAc-3xHA-MLF (2–202), pAc-3xHA-MLF (202–309), pAc-3xHA-MLF (148–309), pAc-3xHA-MLF (96–309), pAc-3xHA-MLF (96–202). DnaJ-1 and MLF cDNA were also cloned into pBlueScript II to generate pBS-DnaJ-1 and pBS-MLF and in pGEX-2T to generate pGEX-DnaJ-1 and pGEX-MLF. All constructs were verified by sequencing.

### Cell culture, dsRNA treatments and transfections

*Drosophila* Kc167 cells were grown at 25°C in Schneider medium (Invitrogen) supplemented with 10% fetal bovine serum (FBS) and 50 μg/ml of penicillin/streptomycin (Invitrogen). For RNAi experiments, double stranded RNA duplexes (dsRNA) corresponding to 400-600bp exonic regions were produced using T7 promoter-containing primers and MEGAscript T7 transcription kit (Ambion). After an annealing step, dsRNA probes were purified using the RNeasy cleanup protocol (Qiagen). Independent dsRNA targeting different regions of *dnaj-1* were produced. The sequences of the T7-containing primers used to generate the dsRNA are available on request. Cells were seeded at 2x10^6^/ml on dsRNA (16 μg/well for 6-well-plate, 8 μg for 12-well-plate and 1 μg for 96-well-plate) and incubated in Schneider medium without FBS for 40 min before being transferred to 5% FBS containing medium. 24h later, cells were transfected with the plasmids of interest using Effectene (Qiagen) and they were collected 72h later for subsequent analyses.

### Luciferase reporter assays

For luciferase assays, 50 ng of *4xPPO2-Firefly* luciferase reporter plasmid, were contransfected with 20 ng of pAc-*Renilla* luciferase plasmid, 10 ng of pAc-Lz-V5 and/or 10 ng of pAc expression plasmid for the protein of interest in 96 well-plate. *Firefly* and *Renilla* luciferases activities were measured 72h after transfection using Promega Dual luciferase reporter assay. Three biological replicates were performed for each transfection assay.

### Real-time quantitative PCR

For RT-qPCR, RNAs were prepared from Kc167 cells using RNeasy kit (Qiagen) with an additional on-column DNAse treatment step. 1 μg of total RNA was used for reverse transcription using Superscript II and random primers (Invitrogen). 10 μl of a 1/300 dilution of cDNA was used as template for real time PCR using HOT Pol Evagreen qPCR mix (Bio-rad). The sequences of the primers used to assess the expression of *dnaj-1*, *mlf*, *lz*, *PPO2*, *Renilla luciferase* and *rp49* are available upon request. All experiments were performed using biological triplicates or quadruplicates.

### *In vitro* pull down assays

pET-3c-Lz, pBS-MLF and pBS-DnaJ-1 plasmids were used as template to produce ^35^S-methionine-labeled proteins *in vitro* using Rabbit Reticulocyte Lysate coupled transcription-translation system (Promega). pGEX-2T, pGEX-MLF and pGEX-DnaJ-1 were used to produce GST, GST-MLF and GST-DnaJ-1 in *Escherichia coli* BL21. Equivalent amounts of GST purified proteins immobilized on Gluthation-Sepharose beads were used to pull down Lz, MLF or DnaJ-1. Proteins were incubated for 2h at 4°C in buffer A (20 mM Tris–HCl, pH 8.0, 150 mM NaCl, 10 mM KCl, 1 mM EDTA, 0.1mg/ml BSA, 1 mM DTT, 0.05% NP40). After extensive washing in buffer buffer B (20 mM Tris-HCl, pH 8.0, 150 mM NaCl, 1 mM EDTA, 1mM DTT, 0.05% NP40), bound proteins were eluted in SDS-loading buffer, separated by SDS–PAGE and visualized by autoradiography.

### Protein extraction, immunoprecipitations and western blots

Kc167 cells were collected, washed in PBS and incubated for 30 min in IP buffer (150 mM NaCl, 0.5% NP40, 50 mM Tris-HCl, pH8.0, 1mM EGTA) supplemented with protease inhibitor cocktail (Roche). The extracts were cleared by centrifugation at 13.000g for 15 min at 4°C and subjected to SDS-PAGE (50 μg of proteins par lane) or immunoprecipitation (1 mg per point). For immunoprecipitation, proteins were preadsorbed with 100 μl of sepharose beads slurry for 1h at 4°C before being incubated with 20 μl of anti-GFP (Chromotek), anti-V5 (Sigma-Aldrich) or anti-HA (Covance) antibody coupled to sepharose beads, or with 10 μl of rabbit anti-MLF [19] or rabbit IgG (SantaCruz) in the presence of 20 μl of protein A sepharose beads (Sigma), for 4h at 4°C. The beads were spun down and washed in IP buffer and immunoprecipitated proteins were processed for SDS-PAGE and Western Blot analyses. Western blots were performed using standard techniques and the blots were developed by photoluminescence procedure using Lumi-Light^PLUS^ Western Blotting Substrate (Roche) and Amersham Hyperfilm^TM^ ECL (GE Healthcare) or Chemidoc Touch Imaging System (BioRad). The following antibodies were used for Western blots: anti-V5 (Invitrogen), anti-HA (BioLegend), anti-GFP, anti-tubulin (Sigma-Aldrich), anti-*Renilla* luciferase (MBL), and anti-MLF [19].

### Affinity purification and mass spectrometry analysis

Stable Kc167 cells carrying an inducible expression vector for MLF were obtained by cotransfecting pMT-MLF-V5-His and pCoBlast (Thermo Fisher Scientific) expression plasmids and selecting individual clones with 25μg/ml blasticidin. For affinity purification, MLF-inducible or parental Kc167 cells were seeded at 10^6^/ml and cultivated for 24h in the presence of 50 mM CuSO4 to induce MLF expression. 20 mg of proteins extracted in IP buffer were then incubated on 200 μl of anti-V5 coupled sepharose beads (Sigma-Aldrich) or 400 μl of anti-V5 coupled magnetic beads (MBL). After several washes in IP buffer, affinity purified proteins were eluted in Laemmli buffer, reduced in 30 mM DTT and alkylated with 90 mM Iodoacetamide before being loaded on 12% SDS-PAGE. The single band of proteins was cut and digested overnight at 37°C with 1 μg of Trypsin (Promega) in 50 mM NH_4_CO_3_. Digested peptides were extracted from the gel by incubating 15 min at 37°C in 50 mM NH_4_CO_3_ and twice for 15 min at 37°C in 5% formic acid/acetonitrile (1:1). The dried peptide extracts were dissolved in 17 μl of 2% acetonitrile, 0.05% trifluoroacetic acid and the peptide mixtures were analyzed by nanoLC-MS/MS using an Ultimate3000-RS system (Dionex) coupled to an LTQ-Orbitrap Velos mass spectrometer (Thermo Fisher Scientific). 5 μl of each peptide extract were loaded on a 300 μm ID x 5 mm PepMap C18 precolumn (LC Packings, Dionex,) at 20 μl/min in 5% acetonitrile, 0.05% trifluoroacetic acid. After 5 minutes desalting, peptides were online separated on a 75 μm ID x 50 cm C18 Reprosil C18 column. The flow rate was set at 300 nl/min. Peptides were eluted using a 0 to 50% linear gradient of solvent B (solvent A: 0.2% formic acid in 5% acetonitrile, solvent B: 0.2% formic acid in 80% acetonitrile) for 80 min at 300nl/min. The LTQ Orbitrap was operated in data-dependent acquisition mode with the XCalibur software (version 2.0 SR2, Thermo Fisher Scientific), on the 350–1800 m/z mass range with the resolution set to a value of 60 000. The twenty most intense ions per survey scan were selected for CID-MS/MS fragmentation and the resulting fragments were analyzed in the linear ion trap (parallel mode). A 60 s dynamic exclusion window was used to prevent repetitive selection of the same peptide. The Mascot Daemon software (version 2.2.0, Matrix Science, London, UK) was used for protein identification against a non-redundant SwissProt database. Mascot results were parsed with Mascot File Parsing and Quantification (MFPaQ) version 4.0 [75]. Quantification of proteins was performed using the label-free module of the MFPaQ software, where a protein abundance index based on the average of peak area values for the three most intense tryptic peptides of the protein was calculated [76]. Triplicate injections were performed.

### RNAseq experiments

RNAseq experiments were performed using independent biological triplicates. For each sample, around 150 third instar larvae of *control* (*lz-GAL4*,*UAS-mCD8GFP/+*) or *mlf* mutant (*lz-GAL4*,*UAS-mCD8GFP/+*, *mlf*^*∂C1*^*/mlf*^*∂C1*^) genotypes were bled in ice-cold PBS. The hemocytes were centrifuged through a 40 μm mesh at 1000 rpm for 1 min and lz>GFP^+^ cells were collected by FACS (FacsAria II) under a pressure of 20 psi. A fraction of the collected cells were used to control GFP^+^ cell purification specificity by examination under an epifluorescent microscope after fixation and mounting in Vectashield medium with DAPI. RNAs were extracted from sorted cells using Arcturus PicoPure RNA kit (Applied Biosystems). RNA samples were run on Agilent Bioanalyzer to assess RNA integrity and concentration. The NuGEN Ovation RNASeq system with Ribo-SPIA technology was used to prepare the cDNA according to the manufacturer instruction. Library preparation was performed using the Illumina TruSeq RNASeq library preparation kit. The resulting libraries were sequenced using a 1x50-bp on Illumina HiSeq 2500. Initial sequence data QC was done using FASTQC. Reads were filtered and trimmed to remove adapter-derived or low quality bases using Trimmomatic and checked again with FASTQC. Illumina reads were aligned to *Drosophila* reference genome (BDGP R5/dm3) with TopHat and Bowtie2. Read counts were generated for each annotated gene using HTSeq-Count. RPKM (Reads Per Kilobase of exon per Megabase of library size) values were calculated using Cufflinks. Read normalization, variance estimation and pair-wise differential expression analysis with multiple testing correction was conducted using the R Bioconductor DESeq2 package. Heatmaps and hierarchical clustering were generated with R Bioconductor. The RNAseq data were deposited on GEO under the accession number GSE93823.

## Supporting information

S1 Fig DnaJ-1 and MLF interact in Kc167 cells. (A, B, C) Western blots showing the results of immunoprecipitation experiments against GFP (A), HA (B) or MLF (C) performed in Kc167 cells transfected with expression vectors for the indicated proteins. (D) Confocal images of fluorescent immunostainings against GFP (green) and HA (red) in Kc167 cells transfected with expression plasmids for GFP-DnaJ-1 and HA-MLF. Nuclei were stained with Topro3. Merged and individual channels are displayed. Scale bar: 10 μm. (E) Autoradiograms showing the results of pull down assays between *in vitro* translated ^35^S-methionine labeled MLF (upper panel) or DnaJ-1 (lower panel) and the indicated GST fusion proteins produced in *E*. *coli*. (F) Western blots showing the results of an immunoprecipitation experiment against GFP in Kc167 cells transfected with expression plasmids for the indicated proteins.
(TIF)

S2 Fig MLF, DnaJ-1 and Hsc70-4 regulate Lz activity. (A-D) Results of RT-qPCR assays showing the relative expression of *mlf*, *dnaj-1*, *lz* and *ppo2* transcripts in Kc167 cells transfected with pAc-Lz-V5 and pAc-Rluc and treated with the indicated dsRNA. (E, F) Luciferase assays (E) and Western blots (F) in Kc167 cells treated with the indicated dsRNA and transfected with 4xPPO2-Fluc reported plasmid in the presence or not (ctr) of pAc-Lz-V5 expression plasmid. pAc-Rluc was used as an internal normalization control. dsHsc70-4 (a) and (b) correspond to two distinct dsRNA targeting Hsc70-4. (G) Autoradiogram showing the results of pull down assays between *in vitro* translated ^35^S-methionine-labeled Lz and the indicated GST fusion proteins produced in *E*. *coli*.
(TIF)

S3 Fig Generation and characterization of *dnaj-1* mutants. (A) Schematic representation of *dnaj-1* locus. *dnaj-1* transcripts and coding sequence (orange) are shown. The location of the sequences targeted by the 2 guide RNAs (gRNA2 and gRNA3), of the P(EPgy2) element used to select CRISPR/Cas9-mediated deletion events, and of the primers (F and R) used for PCR validation are indicated. Part of the region uncovered by the deletion *Def(3L)BSC884* is also indicated. (B) Results of PCR amplification on genomic DNA from wild-type (wt) and putative *dnaj-1* deletion mutants (A, C, D, E and F) using the F and R primers displayed in (A). The mutant lines A and C exhibit a complete deletion of the region located between the two gRNAs, as confirmed by sequencing. Other mutants carried a deletion of *dnaj-1* associated with more complex rearrangements. (C, D) Quantifications of circulating lz>GFP^+^ cell number (C) and size (D) in *lz-GAL4*, *UAS-mCD8-GFP/+* third instar larvae of the indicated genotypes. The *UAS-dnaj-1-∂J* transgene encodes a DnaJ-1 protein deleted for its J-domain. (E, F) Immunostaining against the crystal cell differentiation marker PPO1 was used to assess crystal cell size and number in different *dnaj-1* mutant backgrounds. (E) Relative size of the PPO1^+^ blood cells in bleeds from third instar larvae of the indicated genotypes. (F) Relative number of PPO1^+^ blood cells in bleeds from third instar larvae of the indicated genotypes. (C-F) n.s.: not significant, **: p-value<0.01; ***: p-value<0.001.
(TIF)

S4 Fig MLF expression in Kc167 cells and in larval crystal cells. (A-E) Fluorescent immunostainings against MLF in Kc167 cells (A) or in circulating blood cells from *lz-GAL4*,*UAS-mCD8-GFP/+* control (B), *dnaj1*^*-/-*^ (C), *UAS-dsMLF* (D), and *UAS-dsMLF*; *dnaj1*^*-/-*^ (E) third instar larvae. Nuclei were stained with Topro3. Only MLF staining is shown in the lower panels. Scale bar: 10 μm. (F) Quantifications of MLF level in lz>GFP^+^ circulating blood cells from third instar larvae of the indicated genotypes. *: p-value<0.05, **: p-value<0.01, ***: p-value<0.001.
(TIF)

S5 Fig Notch signaling controls Lz^+^ cell number and size. (A, B) Quantifications of circulating lz>GFP^+^ cell number (A) and size (B) in *lz-GAL4*, *UAS-mCD8-GFP/+* female (left part of the panels) or in *lz-GAL4*, *UAS-mCD8-GFP/Y* male (right part of the panels) third instar larvae of the indicated genotypes. Number and size are relative to control *lz-GAL4*, *UAS-mCD8-GFP/+* females. *: p-value<0.05, **: p-value<0.01, ***: p-value<0.001 as compared to *lz*^*GAL4*^*/+* females (solid lines) or *lz*^*GAL4*^*/Y* males (dashed lines). (C) Representative images of lz>GFP^+^ cells in these different contexts. Scale bar: 10 μm.
(TIF)

S6 Fig MLF and DnaJ-1 repress Notch expression. (A, B) Immunostainings against Notch (NICD: Notch intracellular domain) in blood cells from *lz-GAL4*,*UAS-mCD8-GFP/+* control (A), *mlf*^*-/-*^ (B) and *dnaj-1*^*-/-*^ (C) larvae. NICD staining only is shown in the lower panels. Nuclei were stained with Topro3. (D) Quantifications of NICD immunostainings in lz>GFP^+^ and lz>GFP^-^ blood cells from control, *mlf*^*-/-*^ and *dnaj-1*^*-/-*^ larvae.
(TIF)

S7 Fig Lz represses *Notch* expression. (A) Quantifications of Lz and NICD levels in lz>GFP^+^ circulating blood cells of *lz-GAL4*, *UAS-mCD8-GFP/+* third instar larvae. Cells were pooled into 5 categories according to their size (% of the mean cell size) and Lz or NICD expression level in each pool was plotted. (B-E) Fluorescent immunostainings against GFP and *in situ* hybridizations against *Notch* in circulating blood cells from *lz-GAL4*, *UAS-mCD8-GFP/+* or *lz-GAL4*, *UAS-mCD8-GFP/Y* third instar larvae. Representative images of *Notch* expression in small/medium (B, D) *versus* large (C, E) lz>GFP^+^ cells. Scale bar: 10 μm. Nuclei were stained with Topro3. The lower panels show *Notch* expression only. (F) Schematic representation of the *Notch* locus with the position of the two GMR lines that drive expression in Lz^+^ blood cells. The putative RUNX binding site (red rectangular boxes) and their conservation in different *Drosophila* species are indicated. (G) Lz and GFP expression in *Notch*^*GMR30C01*^*-GAL4*, *UAS-nlsGFP* circulating blood cells from third instar larvae. Nuclei were stained with Topro3.
(TIF)

S1 Table RPKM counts of biological triplicates for all genes in lz>GFP^+^ blood cells from control or *mlf* mutant third instar larvae. (XLSX)

S2 Table List of differentially expressed genes (adjusted *p*<0.01). (XLSX)

S3 Table List of “crystal cell”-associated genes. (XLSX)
