# Supplementary figures and images for: Control of RUNX-induced repression of Notch signaling by MLF and its partner DnaJ-1 during Drosophila hematopoiesis
Source: PLoS Genet. 2017 Jul 25;13(7):e1006932. doi: 10.1371/journal.pgen.1006932 (PMC5549762; doi:10.1371/journal.pgen.1006932)

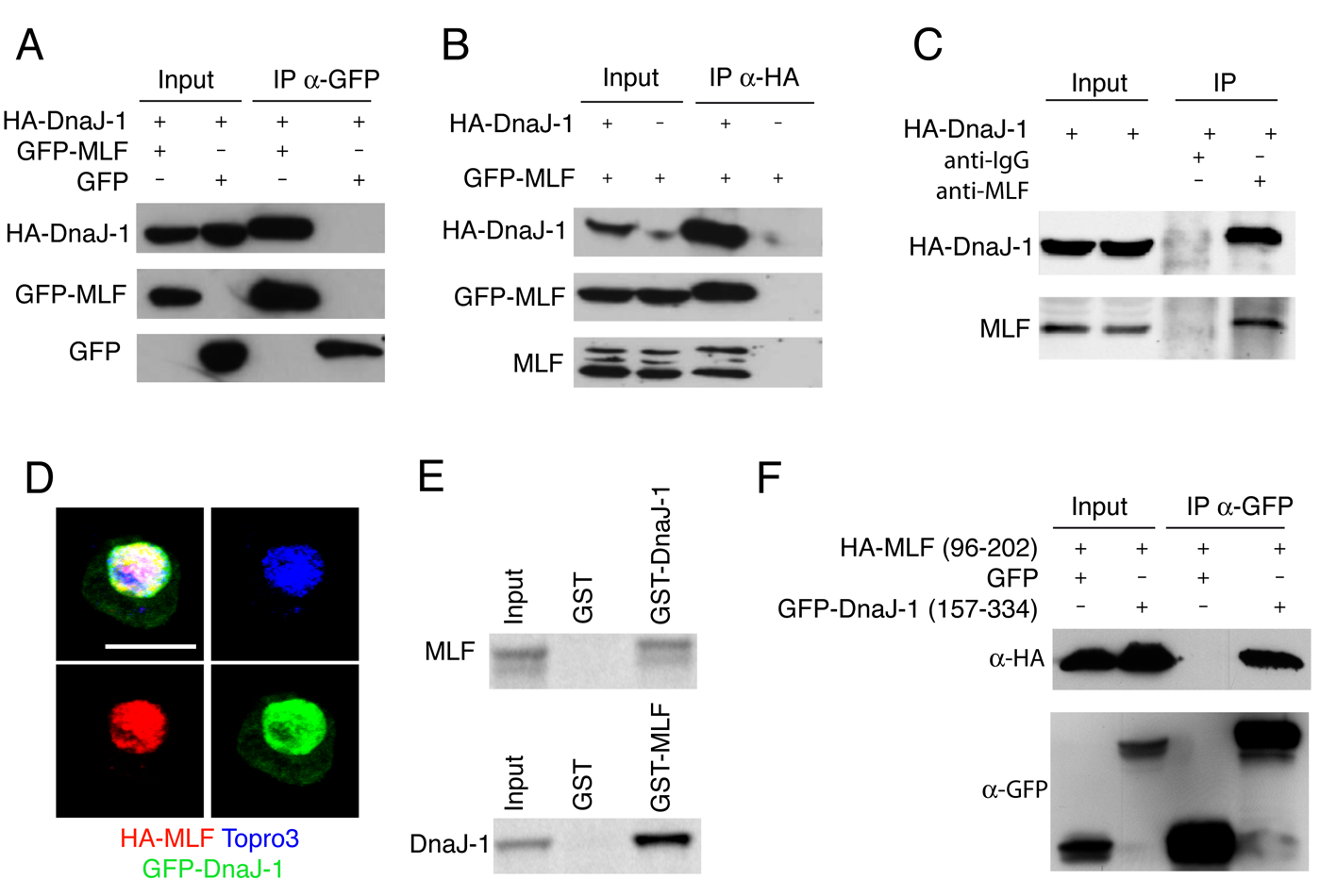

Supplement: S1 Fig — (A, B, C) Western blots showing the results of immunoprecipitation experiments against GFP (A), HA (B) or MLF (C) performed in Kc167 cells transfected with expression vectors for the indicated proteins. (D) Confocal images of fluorescent immunostainings against GFP (green) and HA (red) in Kc167 cells transfected with expression plasmids for GFP-DnaJ-1 and HA-MLF. Nuclei were stained with Topro3. Merged and individual channels are displayed. Scale bar: 10 μm. (E) Autoradiograms showing the results of pull down assays between in vitro translated 35S-methionine labeled MLF (upper panel) or DnaJ-1 (lower panel) and the indicated GST fusion proteins produced in E. coli. (F) Western blots showing the results of an immunoprecipitation experiment against GFP in Kc167 cells transfected with expression plasmids for the indicated proteins. (TIF) [file pgen.1006932.s001.tif]

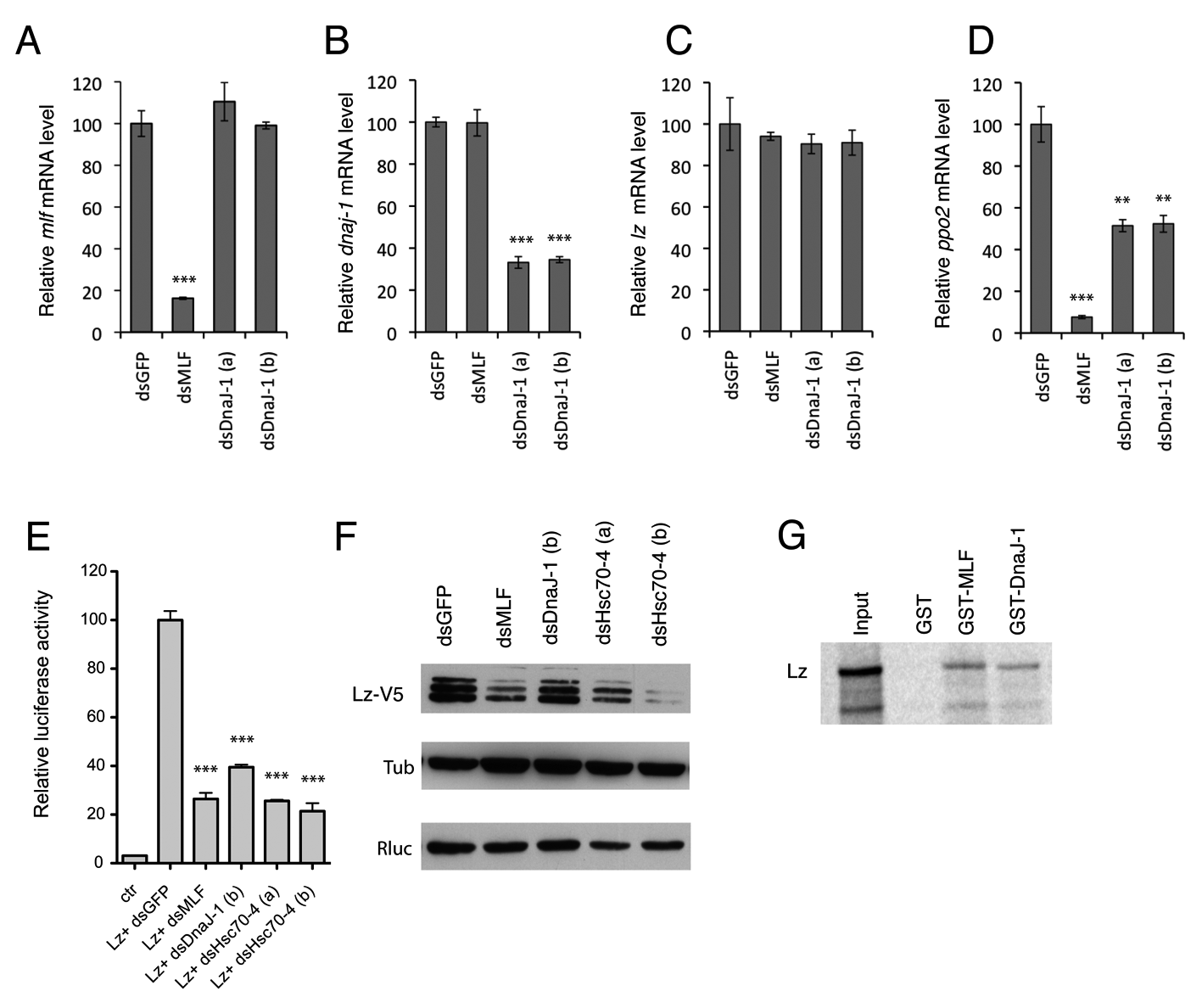

Supplement: S2 Fig — (A-D) Results of RT-qPCR assays showing the relative expression of mlf, dnaj-1, lz and ppo2 transcripts in Kc167 cells transfected with pAc-Lz-V5 and pAc-Rluc and treated with the indicated dsRNA. (E, F) Luciferase assays (E) and Western blots (F) in Kc167 cells treated with the indicated dsRNA and transfected with 4xPPO2-Fluc reported plasmid in the presence or not (ctr) of pAc-Lz-V5 expression plasmid. pAc-Rluc was used as an internal normalization control. dsHsc70-4 (a) and (b) correspond to two distinct dsRNA targeting Hsc70-4. (G) Autoradiogram showing the results of pull down assays between in vitro translated 35S-methionine-labeled Lz and the indicated GST fusion proteins produced in E. coli. (TIF) [file pgen.1006932.s002.tif]

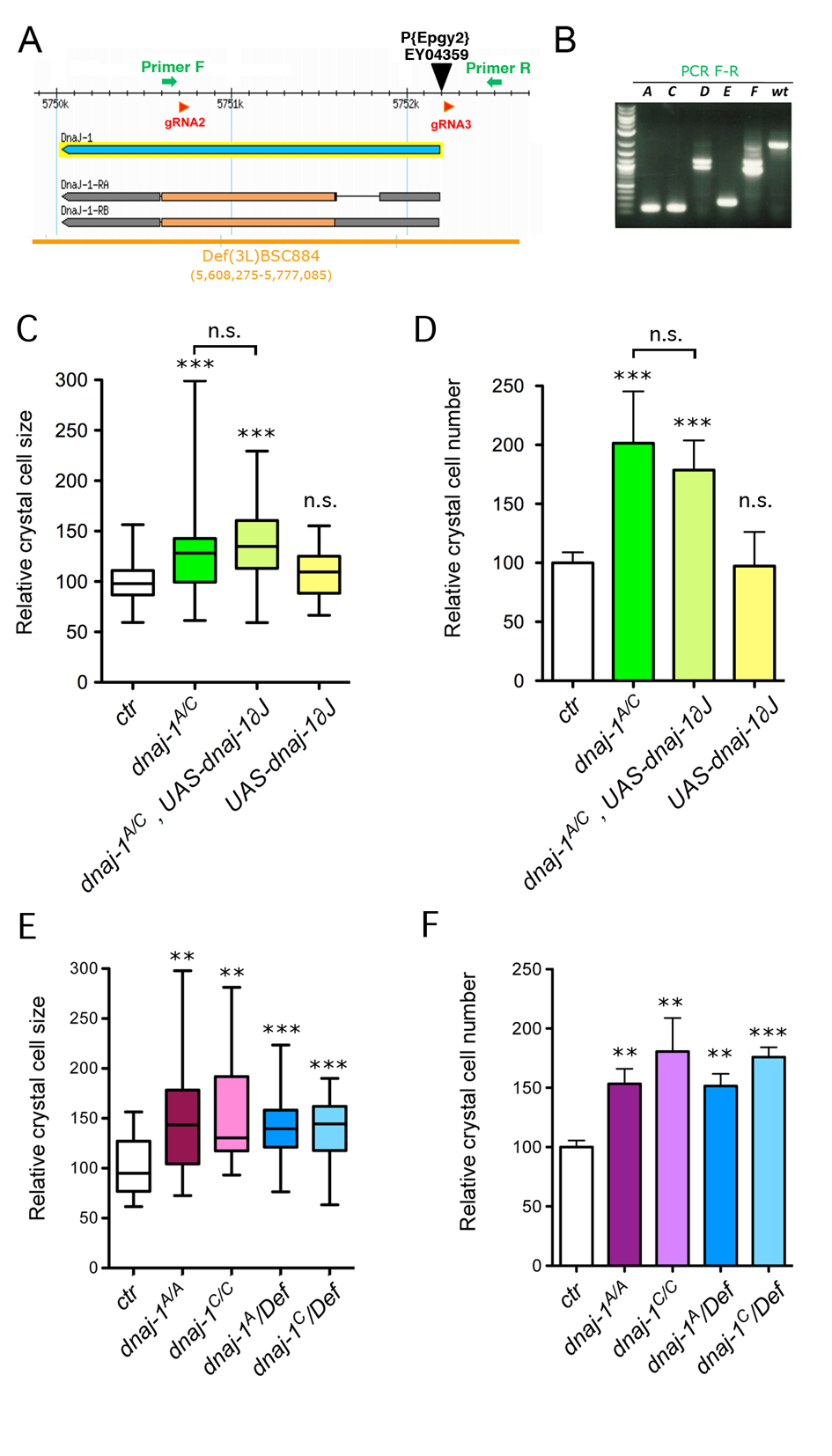

Supplement: S3 Fig — (A) Schematic representation of dnaj-1 locus. dnaj-1 transcripts and coding sequence (orange) are shown. The location of the sequences targeted by the 2 guide RNAs (gRNA2 and gRNA3), of the P(EPgy2) element used to select CRISPR/Cas9-mediated deletion events, and of the primers (F and R) used for PCR validation are indicated. Part of the region uncovered by the deletion Def(3L)BSC884 is also indicated. (B) Results of PCR amplification on genomic DNA from wild-type (wt) and putative dnaj-1 deletion mutants (A, C, D, E and F) using the F and R primers displayed in (A). The mutant lines A and C exhibit a complete deletion of the region located between the two gRNAs, as confirmed by sequencing. Other mutants carried a deletion of dnaj-1 associated with more complex rearrangements. (C, D) Quantifications of circulating lz>GFP+ cell number (C) and size (D) in lz-GAL4, UAS-mCD8-GFP/+ third instar larvae of the indicated genotypes. The UAS-dnaj-1-∂J transgene encodes a DnaJ-1 protein deleted for its J-domain. (E, F) Immunostaining against the crystal cell differentiation marker PPO1 was used to assess crystal cell size and number in different dnaj-1 mutant backgrounds. (E) Relative size of the PPO1+ blood cells in bleeds from third instar larvae of the indicated genotypes. (F) Relative number of PPO1+ blood cells in bleeds from third instar larvae of the indicated genotypes. (C-F) n.s.: not significant, **: p-value<0.01; ***: p-value<0.001. (TIF) [file pgen.1006932.s003.tif]

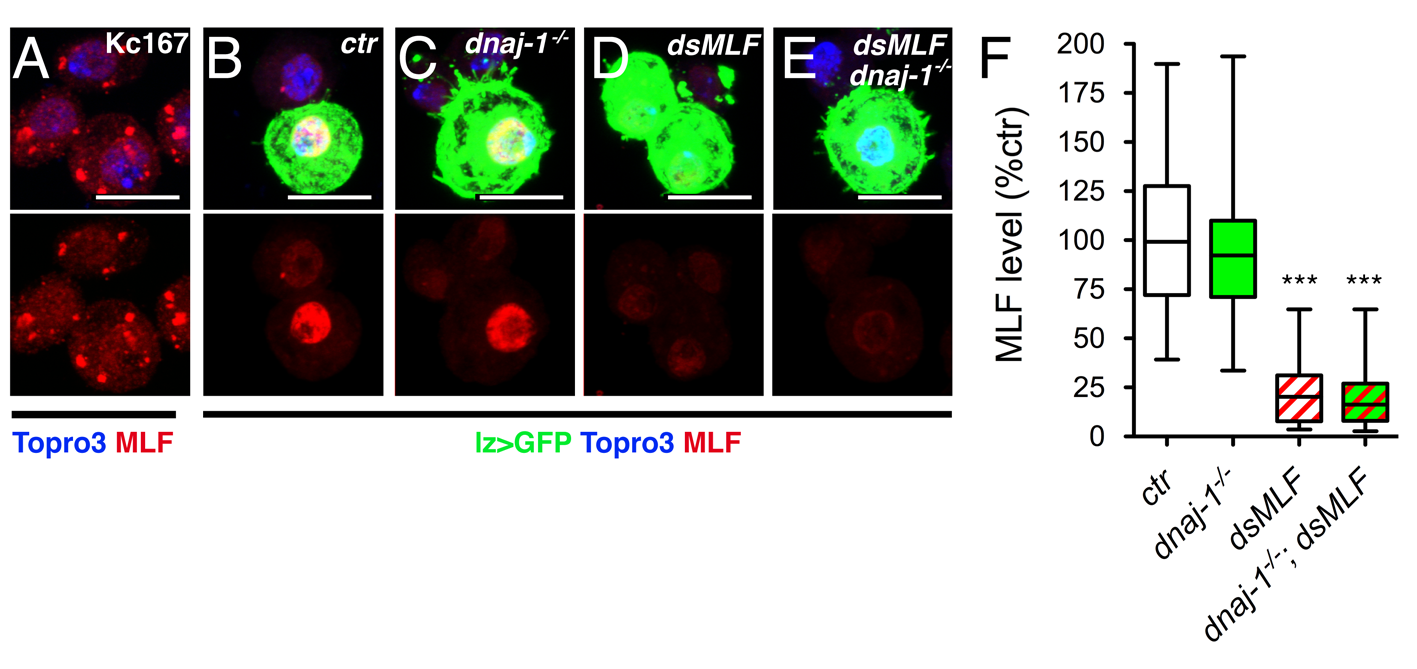

Supplement: S4 Fig — (A-E) Fluorescent immunostainings against MLF in Kc167 cells (A) or in circulating blood cells from lz-GAL4,UAS-mCD8-GFP/+ control (B), dnaj1-/- (C), UAS-dsMLF (D), and UAS-dsMLF; dnaj1-/- (E) third instar larvae. Nuclei were stained with Topro3. Only MLF staining is shown in the lower panels. Scale bar: 10 μm. (F) Quantifications of MLF level in lz>GFP+ circulating blood cells from third instar larvae of the indicated genotypes. *: p-value<0.05, **: p-value<0.01, ***: p-value<0.001. (TIF) [file pgen.1006932.s004.tif]

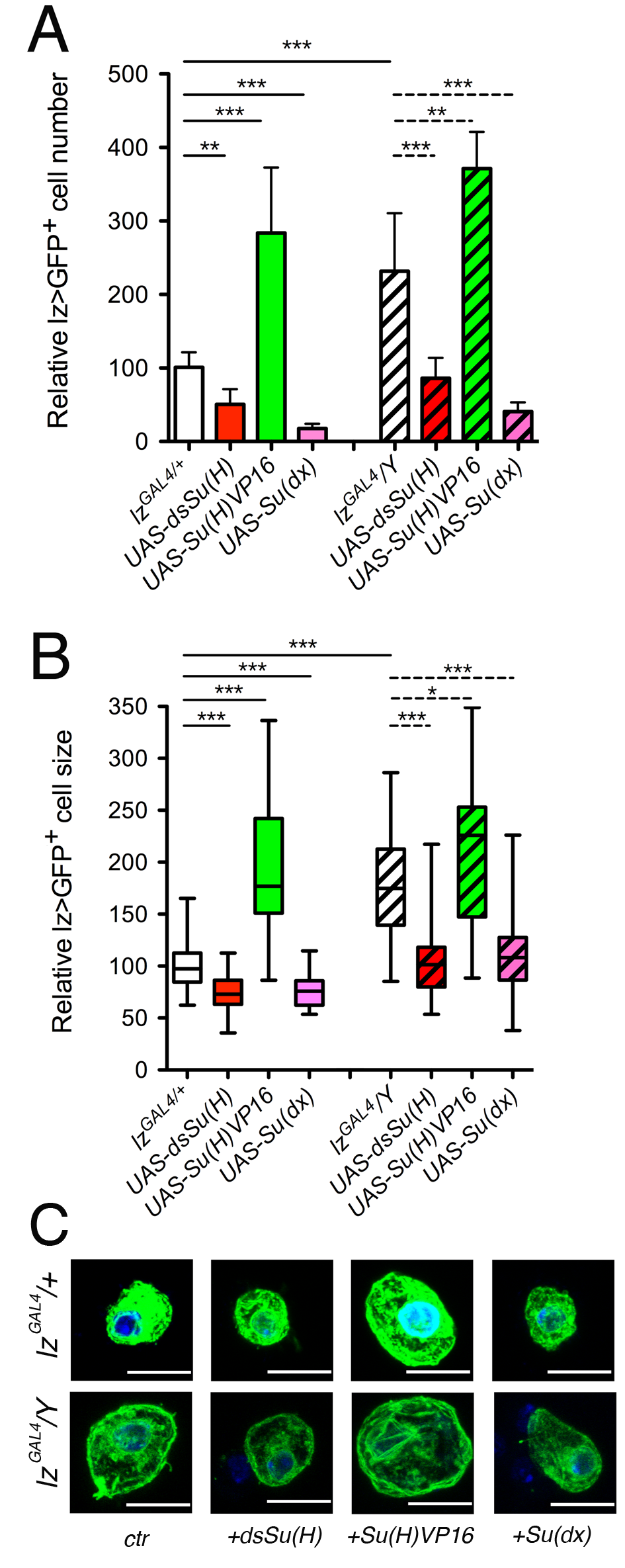

Supplement: S5 Fig — (A, B) Quantifications of circulating lz>GFP+ cell number (A) and size (B) in lz-GAL4, UAS-mCD8-GFP/+ female (left part of the panels) or in lz-GAL4, UAS-mCD8-GFP/Y male (right part of the panels) third instar larvae of the indicated genotypes. Number and size are relative to control lz-GAL4, UAS-mCD8-GFP/+ females. *: p-value<0.05, **: p-value<0.01, ***: p-value<0.001 as compared to lzGAL4/+ females (solid lines) or lzGAL4/Y males (dashed lines). (C) Representative images of lz>GFP+ cells in these different contexts. Scale bar: 10 μm. (TIF) [file pgen.1006932.s005.tif]

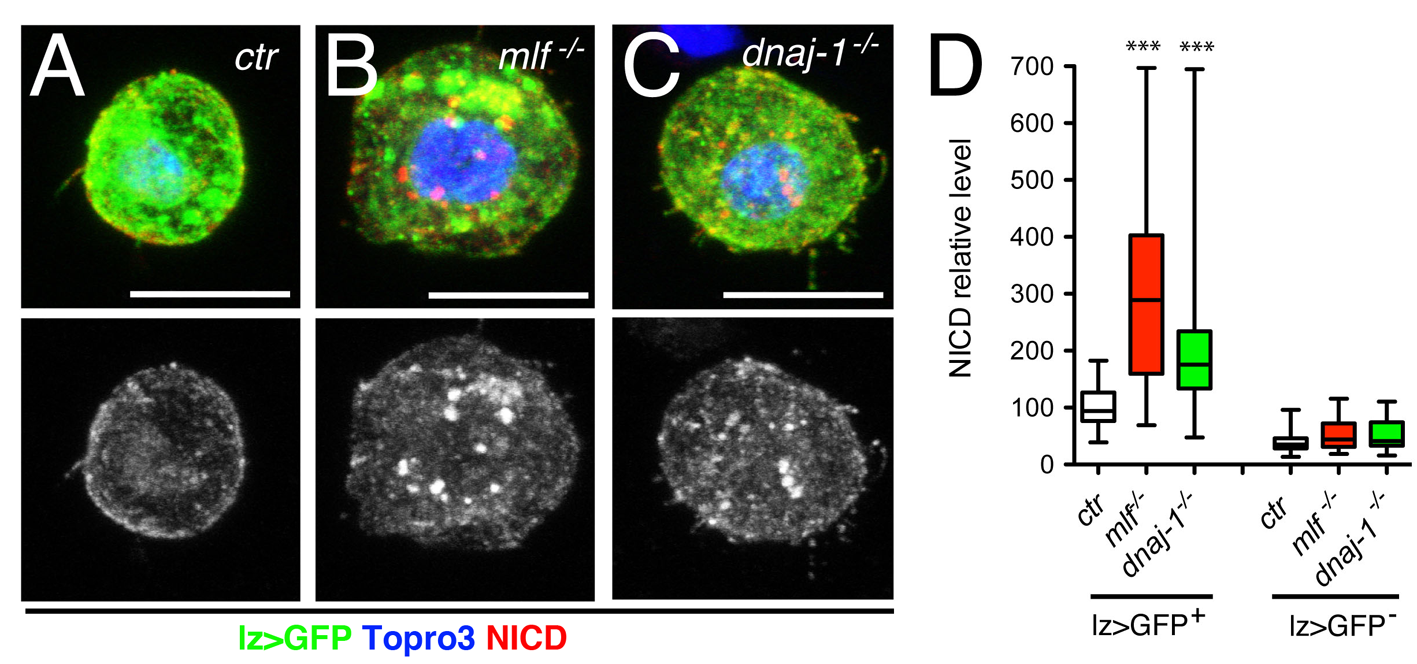

Supplement: S6 Fig — (A, B) Immunostainings against Notch (NICD: Notch intracellular domain) in blood cells from lz-GAL4,UAS-mCD8-GFP/+ control (A), mlf-/- (B) and dnaj-1-/- (C) larvae. NICD staining only is shown in the lower panels. Nuclei were stained with Topro3. (D) Quantifications of NICD immunostainings in lz>GFP+ and lz>GFP- blood cells from control, mlf-/- and dnaj-1-/- larvae. (TIF) [file pgen.1006932.s006.tif]

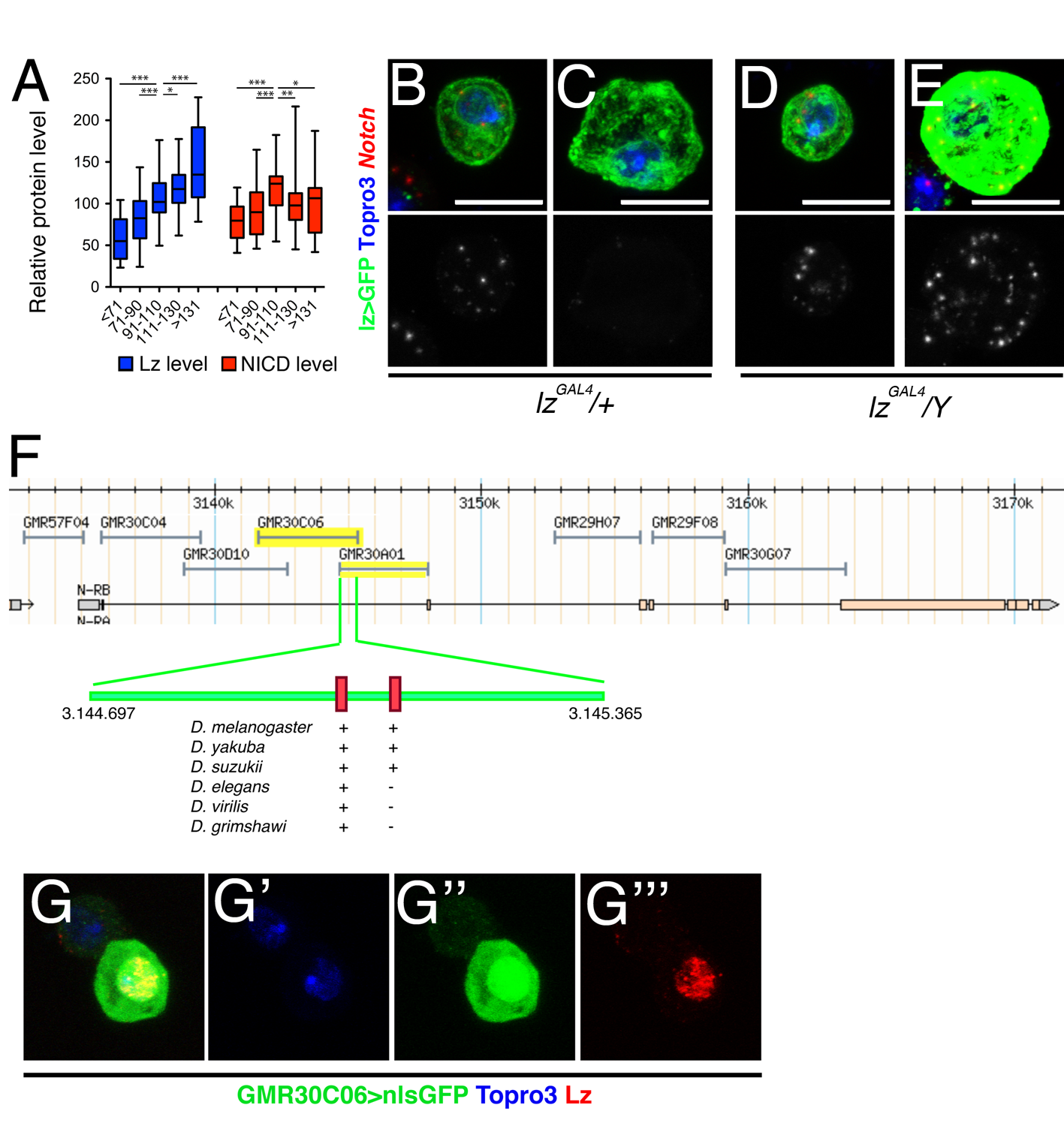

Supplement: S7 Fig — (A) Quantifications of Lz and NICD levels in lz>GFP+ circulating blood cells of lz-GAL4, UAS-mCD8-GFP/+ third instar larvae. Cells were pooled into 5 categories according to their size (% of the mean cell size) and Lz or NICD expression level in each pool was plotted. (B-E) Fluorescent immunostainings against GFP and in situ hybridizations against Notch in circulating blood cells from lz-GAL4, UAS-mCD8-GFP/+ or lz-GAL4, UAS-mCD8-GFP/Y third instar larvae. Representative images of Notch expression in small/medium (B, D) versus large (C, E) lz>GFP+ cells. Scale bar: 10 μm. Nuclei were stained with Topro3. The lower panels show Notch expression only. (F) Schematic representation of the Notch locus with the position of the two GMR lines that drive expression in Lz+ blood cells. The putative RUNX binding site (red rectangular boxes) and their conservation in different Drosophila species are indicated. (G) Lz and GFP expression in NotchGMR30C01-GAL4, UAS-nlsGFP circulating blood cells from third instar larvae. Nuclei were stained with Topro3. (TIF) [file pgen.1006932.s007.tif]
